# Rethinking revolving door research: a scoping review of methods and datasets used by non-academics to examine the revolving door

**DOI:** 10.1186/s12992-025-01184-7

**Published:** 2026-01-16

**Authors:** Saskia Jaenecke, Jennifer Lacy-Nichols

**Affiliations:** Centre for Health Equity, Melbourne School of Population and Global Health, University of Melbourne, Level 4, 207 Bouverie St, Victoria, 3010 Australia

**Keywords:** Revolving door, Corporate political activity, Commercial determinants of health, Political determinants, Data, Methods

## Abstract

**Background:**

Business lobbying is a risk to public health. Evidence demonstrates that corporations seek to block, delay and otherwise undermine the development and implementation of policies that they view as threatening to their profits (such as warning labels on alcohol or tobacco taxes). Yet, corporate political influence has proven immensely challenging to study, with data often missing, incomplete or fragmented. This paper is part of a larger research program that seeks to develop methods to analyse corporate political activities. In this paper, we explore different approaches and datasets used by non-academics to examine the revolving door: the movement of individuals between public and private sector employment. To date, there is limited public health research on this topic. Our aims were twofold: first, to understand what aspects of the revolving door have been explored outside academia, and second, to understand how useable and reuseable the datasets and resources were.

**Methods:**

We conducted a systematic scoping review of grey literature in Australia, the United States and United Kingdom. We developed a simple framework to assess six features of revolving door data use and reuse: downloadable; interactive features; dates; job titles and employers; context; and dataset size.

**Results:**

We identified 41 records: 17 from Australia, 10 from the UK and 14 from the USA. Most originated from non-governmental organisations, followed by media and government. Of the records that focused on a specific industry, fossil fuels were most common followed by the defence and weapons sector. Only three records demonstrated a high level of data use or reuse.

**Conclusions:**

Our study highlights a range of different data sources that could be explored in future studies as well as strategies to make data more reusable. It also reveals some of the challenges of studying the revolving door, including intellectual property and privacy laws. As public health researchers increasingly study the political activities of businesses, they must carefully consider how to balance efforts to make data more accessible for policymakers and advocates, while not unfairly compromising individual privacy.

**Supplementary Information:**

The online version contains supplementary material available at 10.1186/s12992-025-01184-7.

## Introduction

Comparative research suggests that countries with greater levels of corporate political influence have lower levels of implementation of WHO recommendations for the ‘best buy’ policies to prevent non-communicable diseases [1]. This is a quintessential example of how commercial political influence can undermine public health progress. Tobacco industry lawsuits in the international courts have deterred countries from implementing tobacco plain packaging laws [2, 3]. Alcohol lobbying has delayed implementation of key harm reduction policies [4, 5]. As these examples demonstrate, corporate political influence can take many forms, including campaign donations, hiring former politicians as lobbyists or commercial actors sitting on government committees. While it is rare (if not highly unlikely) for any singular activity to be the ‘smoking gun’ that shapes political decision-making, taken together, corporate political activities create an environment in which public health – and other public interest – policies and legislation face significant, costly and sometimes insurmountable opposition from commercial interests [2, 4, 6, 7].

Documenting, disentangling, interrogating and understanding the myriad facets of corporate political influence is a crucial public health advocacy task. Yet understanding and measuring corporate political influence is immensely challenging. One challenge is practical: data are thin on the ground. Government disclosures of political activities are relatively rare internationally, and even in countries with robust data (the United States, for instance), it is only through the time- and resource-intensive efforts of government agencies and non-governmental organisations that the data is accessible and easy to use [8, 9]). The second challenge is conceptual: political influence – and power more broadly – is complex, abstract and slippery. Indeed, Dahl described the efforts to define and describe power in political theory as “giant globs of oily ambiguity” [10]. To state the challenge simply: we lack consensus on what is important to measure, and often, we can’t measure it anyways. In this paper, we tackle the practical question of data availability for one form of corporate political influence that has proven especially challenging to study: the revolving door.

### The revolving door and conflicts of interest

The revolving door refers to the movement of individuals between public and private sector employment – often from policy roles into government relations or lobbying. Revolving ‘out’ (e.g. politicians turned lobbyists) allows commercial organisations to leverage relationships and insider knowledge of government processes for undue influence; likewise, revolving ‘in’ (commercial actors moving into government) can embed an industry-favourable mindset within policymaking and regulation [11]. While the revolving door does provide benefits (expanding skills and experience for government and private sector), without effective safeguards it risks corporate interference in public interest policies. For example, a study of tobacco industry lobbyists found almost half had held position in Australian governments before or after working in the tobacco industry, raising concerns about their ability to access government officials and lobby for weaker regulations of e-cigarettes [12].

While media attention to the revolving door often focuses on sensational cases of high-ranking members of government moving into lucrative private sector positions, the revolving door occurs at all levels of government (more often, in fact, in the bureaucracy) [13]. Definitions of the revolving door differ in their breadth. Some focus more narrowly on the movement of former members of government (and their staff) into (and from) government relations roles where they could seek to influence public policy decisions [14, 15]). Others take a far broader lens capturing the passage between public and private sector employment writ large. While the revolving door can offer a range of benefits (expanding the expertise and experience of the public and private sectors), it also raises concerns about the potential for public interests becoming subservient to private.

One concern centres on the corporate ‘capture’ of regulatory agencies. This risk is especially visible in the area of drug approvals and regulations, where it is common for regulators to move into roles working for the companies they used to regulate (or to come from those companies) [16]. For example, a former president of Eli Lilly (a multinational pharmaceutical company) was appointed as the US Secretary of Health and Human Services, and a Pfizer employee moved to the US Food and Drug Administration (FDA) (responsible for drug approvals) to work as a regulator – after which they rejoined Pfizer as their chief medical officer [17, 18]. These are not isolated examples. Researchers estimate that almost a one-third of people appointed to the Department of Health and Human Services in the United States moved into industry positions at the end of their appointment [19] The close connections between regulators and drug companies (or other industry sectors) raise concerns about the integrity of government decision-making when regulators have an industry-favourable mindset (either because they have come from industry or aim to go into it). In the context of the opioid crisis in the US, documents subpoenaed from Purdue Pharma about OxyContin (a painkiller) suggest that one of the FDA regulators who led the 1995 approval for OxyContin “might have” drafted the drug label text which allowed OxyContin to be marketed as less addictive and suitable for general cases of pain [16]. Approximately one year after leaving the FDA, this regulator took up a position with Purdue [16].

A second concern focuses on the potential for private interests to exert an undue or excessive influence over government decision-making via the revolving door. In this context, a ‘revolver’ has left government and taken a position in the private sector as a lobbyist, consultant, lawyer or other role where their connections into government are valuable. Political science scholarship has explored the question of the value of revolving door lobbyists, asking if it is their policy and procedural expertise or if it is their access and connections into government – put simply, is it what or whom they know [20]. Scholars conclude it is both, and that the policy issue and context determines which is prioritised [13, 20]. Studies exploring the impact of the revolving door have found that hiring a revolving door lobbyists increases the likelihood of lobbying success [21], for instance, pharmaceutical companies which hire former FDA officials have higher rates of drug approvals [22]. Indeed, the importance of government connections and political experience is materially evidenced with research showing that revolving door lobbyists are paid a premium [23]. Regardless of the source of influence, the concern here is that revolving door lobbyists provide an advantage to their commercial employers that is financially out of reach for individual citizens or public interest groups, ultimately risking policymaking prioritising private interests over the public. 

To prevent and mitigate these concerns, many countries have developed policies and legislation to minimise conflicts of interest and help ensure governments act in the public interest and remain accountable. One approach focuses on transparency, such as ‘Sunlight’ laws and disclosure requirements (e.g. lobbyist registers that requires lobbyists to declare if they have previously worked in government) [24–26]. A second approach regulates post-government employment. A common regulatory mechanism is cooling-off periods, which restrict former members of government from moving directly into lobbying roles (or other forms of private sector employment) [11]. Other mechanisms include oversight groups that review potential employment opportunities and advise on how conflicts of interest should be addressed [11].

While these measures are useful in principle, a common criticism is that they lack implementation, enforcement and monitoring, rending them weak and ineffective [11, 24]. Further, a narrow definition of lobbying activity can severely limit the effectiveness of lobbying regulations. For example, in Australia, a narrow definition of ‘lobbyist’ in the code of conduct means that in-house lobbyists (e.g. government relations) are excluded [27]. This effectively allows former government officials to move directly into a wide variety of roles within the very industries they used to regulate [11]. Weak regulation of and transparency around the revolving door presents a risk that harmful industries can gain privileged access to government decision-making processes – and hide it.

### Efforts to expose, study and monitor the revolving door

One of the most persistent themes in revolving door research is that research is challenging. Data is scarce, fragmented and incomplete. In one of the seminal studies of the revolving door, the authors describe their search process as ‘time-consuming and laborious,’ noting that three research assistants spent the better part of a year triangulating multiple data sources to develop employment histories for 1600 lobbyists (just 11% of the full sample of lobbyists) [13]. Amongst the many findings from this study, the authors found that two-thirds of revolving door lobbyists failed to report information about previous government employment – almost 4000 lobbyists if extrapolated to the full 2008 sample.

Despite these challenges, there is a rich body of literature exploring the revolving door. Researchers have explored the population of revolving door lobbyists [28, 29], the different types of revolving door lobbyists [14, 30, 31], the circumstances under which revolving door lobbyists are valuable [32], and the impact of revolving door lobbyists on policy [20, 33, 34]. These studies sit largely in the discipline of political science, which takes a macro view seeking to explain patterns in the lobbying ecosystem.

In contrast, the revolving door has received scant attention from public health scholars. Some studies of commercial determinants of health have included the revolving door, but not conducted a substantive empirical analysis [35–38]. In our Australian context, we are aware of just five academic studies explicitly analysing the revolving door [12, 30, 39–41] Only two of these bring a public health perspective to the topic, looking at the tobacco industry and professional lobbyists working for the alcohol, tobacco, gambling and food industries [12, 41].

Given the time-consuming nature of collecting, cleaning, coding and analysing revolving door data, we were interested to identify approaches to studying the revolving door that generated reusable data. Non-academic approaches offer new possibilities for collecting, analysing and sharing information. A wide variety of individual exposés and case studies documented by investigative journalists, advocacy groups, thinks tanks and other types of non-government organisations (NGOs) offer insights and additional information about the revolving door. While many of these focus on individual, high-profile cases, a substantial number undertake comprehensive data collection and analysis of revolving door activities. The non-governmental organisation (NGO) OpenSecrets in the United States, for example, has perhaps the largest dataset of revolving door activities internationally [15].

Considering that data is perhaps the single largest challenge for revolving door research, this paper explores approaches to documenting and investigating the revolving door used by non-academics (e.g. journalists, NGOs) that generate reusable data. Our aims were twofold: first, to understand what aspects of the revolving door have been explored outside academia, and second, to understand how useable and reuseable the datasets and resources were produced by these groups. This latter aim is especially central to our wider research program, which seeks to advance methods to understand and monitor corporate political activities. By exploring different approaches to investigating the revolving door in different contexts and by different groups, we seek to expand our collective research ‘toolbox’.

## Methods

We conducted a scoping review of grey literature, combining the global standard for conducting scoping reviews outlined in Arskey and O’Malley [42] with techniques from Godin et al. [43] for systematically exploring grey literature. A scoping review is suitable as it helps to map out key concepts and methods and can identify research gaps [44]. Our review was structured into five key stages: (1) research question identification; (2) conceptual search strategy and keyword development; (3) inclusion criteria; (4) literature search and screening; (5) data charting and analysis. Our research questions asked how the revolving door has been investigated outside academia, and how useable and reusable are the data that are generated.

In addition to mapping approaches used to investigate the revolving door in the Australian context, we also explored approaches used in the United Kingdom and United States. As revolving door data often comes from government data sources (e.g. lobbyist registers), we chose the UK for its comparable political context [45] and media system [46] to Australia. The USA was included as it is considered to have a high level of transparency and disclosures regarding political activities [24], meaning these data sources are likely to be some of the richest.

### Search strategy and keyword development

Following Godin et al.’s approach, we developed a series of conceptual categories for the revolving door based on our analysis of the literature and piloting of different search terms using Google’s advanced search tool. As the term ‘revolving door’ can refer to both physical revolving doors and the political concept, we developed four conceptual categories (commercial, government, lobbying, conflict of interest) that signalled a political aspect of the term. We also developed a fifth category for more general terms related to employment. In contrast to database searches where a single search strategy might suffice, Google searches sometimes “require creating several search strategies containing multiple combinations of search terms” [43]. Each of our keyword search strings combined the term ‘revolving door’ with one of the political categories. We also combined these political categories alone and with the ‘employment’ category to capture relevant resources that might not explicitly refer to the ‘revolving door’ but instead describe the phenomenon in other ways. Supplementary File 1 details our search strategies.

### Inclusion criteria

We applied six inclusion criteria to our review (Table 1). In line with RQ 2, we developed two criteria as proxies for useable and/or reusable data. Our first proxy was the size of the dataset. To develop this criterion, we documented the number of revolving door ‘cases’ from a sample of records from Google Searches in the Australian context. Following this, we decided on a threshold of 10 for the size of the dataset, as this allowed us to include some seminal resources that had fewer cases. Consequently, more ad hoc media articles including a single revolving door case were excluded, as these types of resources did not offer a dataset per se. Our second proxy focused on how well-organised the data was, documenting the different ways that the revolving door cases were presented and how easy it was to quickly identify them (or preferably, extract them for future research). While a downloadable spreadsheet was considered the ideal case, as few sources provided these, we also included documents where information was organised or presented in a way to facilitate its reuse (for example, relevant text bolded in media articles).

**Table 1:** Inclusion and exclusion criteria

Inclusion	Exclusion
Written format	Other non-written formats including radio segments or interviews
Written in the English language	Written in languages other than English
Grey literature material including media reports, policy documents, or civil society or industry publications or webpages	Commercially published (e.g. books) or peer-reviewed academic material
Documented cases of the revolving door, or movement between the public and private sector, at individual, company, industry or government level	Purely informative or recommendation-oriented content that does not include documented cases of the revolving door
10 or more units of analysis, which could be: • Number of individual revolving door cases • Number of cases per company • Percentages of lobbyists with a revolving door history	Less than 10 units of analysis, which could be: • Number of individual revolving door cases • Number of cases per company • Percentages of lobbyists with a revolving door history
Data or information is organised, formatted, or presented in a manner that is easily able to be extracted and reused, including: • Databases • Spreadsheets • Lists or tables • Graphs or figures • Highlighted or bold text	Data or information is not organised, formatted or presented in a manner that is easily able to be extracted and reused, including: • Purely narrative text without structured data • Isolated or singular data points

### Literature search and screening

**Fig. 1:**
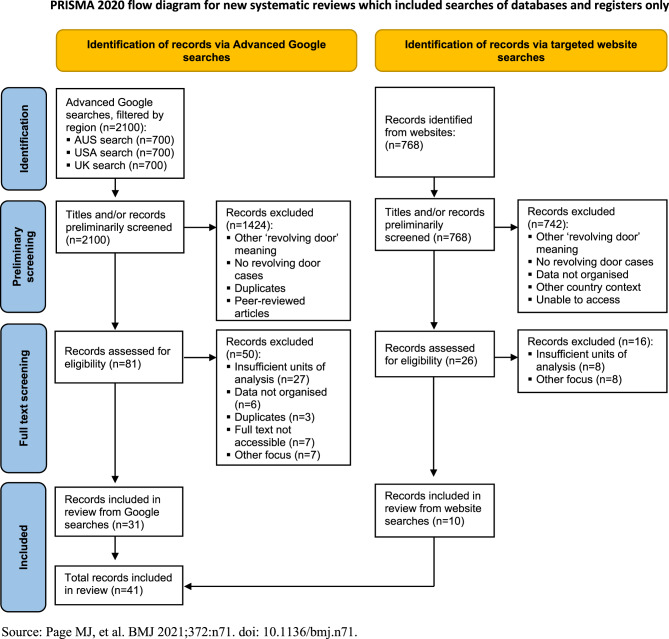
PRISMA flow diagram

We conducted 21 Advanced Google searches filtered by each region (seven each in Australia, USA, UK, corresponding to the different keyword combinations we developed – see Supplementary file 1). The first 100 results for each search were screened (as is standard in grey literature reviews) – totalling 2100 results across all 21 searches. Following our Google searches, to identify any further resources not yet captured, we conducted targeted searches of the term ‘revolving door’ across relevant Australia, USA and UK and international organisation websites. The list of organisations we searched was based on JLN’s expertise as well as collections from previous scoping studies [47–49]. For websites where a search function was available, the term ‘revolving door’ was entered, and for websites without, research repositories or publications were manually scanned. Searches were carried out between August 2024 and March 2025. This search process is documented in further detail within Supplementary File 1 and Fig. 1.

As abstracts are often unavailable within grey literature, other elements such as tables of contents or executive summaries were initially used for preliminary screening, as outlined by Godin et al. [43] Due to the wide range of formats, in other cases records required a full scan to determine their relevance. These cases were documented to avoid duplicating efforts during full screening. Our inclusion and exclusion criteria guided our preliminary and full-text screening. Any discrepancies were discussed and reviewed by both authors to ensure they met our inclusion criteria. Table 4 in Supplementary File 1 lists all results excluded during full screening.

### Data charting and analysis

To extract our data, we used the technique of charting, as is common in scoping reviews, to identify, sift and sort information from each record [42]. This method allowed us to identify patterns in regard to how the revolving door had been previously investigated. Charted characteristics continued to be refined throughout the extraction and analysis process, as this is often regarded as an iterative process within scoping reviews [50].

Our research questions guided the development of our charting framework. For our first research question, we charted the following categories: record details (country, year, sector, organisation, title); industry focus; type of data (units of analysis, number of units); and methods (description of methodology and data sources). We also documented challenges or recommendations identified in each record as well as information about the organisation’s funding sources and its purpose (these are presented in Supplementary File 2 along with additional details about units of analysis).

To assess levels of data use and reuse (our second research question), we developed and charted six criteria for each record: (1) downloadable data, (2) interactive features, (3) dates, (4) job titles and employers, (5) context and (6) dataset size. All criteria were evaluated for each record and awarded points based on whether they did not (0pts), partially (0.5pts) or fully met (1pt) the criteria, with a tallied score presented for each resource (Supplementary File 1 details the complete scoring matrix). We present our results in a heatmap to show which resources did not (light coloured), partially (medium) or fully met (dark) each criterion.

## Results

### How has the revolving door been analysed?

We identified a total of 41 grey literature resources (both reports and webpages) (Table 2). Of these, 17 (41%) were from Australia, 10 (24%) from the UK, and 14 (34%) from the USA. The earliest publication was in 2003 and the most recent was 2024 (no time limit was placed on our searches). The resources we included stemmed from a range of organisations. Most originated from not-for-profit or non-governmental organisations (NGO) (23, 56%), followed by media (12, 29%), government (4, 10%), and academic (2, 5%). These organisations focused on a wide range of issues including the environment and human rights. Most of the NGOs had an interest in public policy or government integrity (see Supplementary File 2). Most resources did not have an industry-specific focus, focusing on lobbying/revolving door on a general level (22, 53%). For those resources that did focus on specific industries, the most prevalent was fossil fuels (11, 27%), followed by military and weapons (8, 20%). We note that several records focused on more than one industry, so the total number of industries analysed was greater than our record total. We assigned each record a unique ID in Table 2 (e.g. S1), which are used to report our findings below.

**Table 2:** Revolving door resources

Ref #	Country	Year	Sector	Organisation	Title	Industry focus	Units of analysis	Methodology described	Data sources described
**S1**	AUS	2023	Media	Michael West Media	Revolving Doors – Democracy at risk	Fossil fuels; Military; Financial Services; Games & Liquor	Individual; Industry; Government	None	None. They note that the identities of their sources are guarded to ensure their absolute confidentiality
**S2**	AUS	2024	NFP	Centre for Public Integrity	The Lobbyist Register	Lobbying general	Individual	Limited	Australian Government Register of Lobbyists
**S3**	AUS	2024	Media	Crikey	The gravy train list	Revolving door general	Individual; Government	None	Hyperlinks for some
**S4**	AUS	2016	NFP	Australia Institute & Australian Conservation Foundation	Greasing the Wheels	Fossil fuels	Individual	None	LinkedIn and other referenced webpages
**S5**	AUS	2024	NFPs	Our Democracy Alliance (Australian Conservation Foundation, the Human Rights Law Centre and the Australian Democracy Network)	Explainer: It’s time to shine a light	Gambling; Military; Fossil fuels; Tobacco	Individual	None	Reference list included. Range of sources linked from media, organisational, academic, codes of conduct
**S6**	AUS	2019	NFP; Media	Greenpeace & Michael West	Dirty Power	Fossil fuels	Individual; Company; Industry	Limited	References included throughout report, though not within the connections diagram itself.
**S7**	AUS	2018	Media	The Conversation	Revealed: the extent of job-swapping	Fossil fuels	Company; Government	None	Hyperlinks for some
**S8**	AUS	2024	Media	The Klaxon	Lockheed Martin, Australian Government joined at hip	Military/Arms	Individual; Company	None	Hyperlinks for some
**S9**	AUS	2023	NFP	Centre for Public Integrity	Closing the revolving door	Lobbying general	Individual	None	Reference list included. Range of sources linked from media, organisational, academic, codes of conduct
**S10**	AUS	2024	Media	Mandarin & Crikey	The Mandarin and Crikey’s ‘revolving door’ list	Consulting	Individual; Company	None	Links included for each person, e.g. LinkedIn, or company websites.
**S11**	AUS	2024	Media	Crikey	Advisory firms to big pharma	Revolving door general	Individual	None	Hyperlinks for some
**S12**	AUS	2020	NFP	350 Perth	Captured State	Fossil fuels	Individual; Company; Government	Limited	RD info: public LinkedIn profiles and ministerial staff records. Other: FOI requests; publicly available information on company and government websites; Annual Donor Returns on the Australian Electoral Commission; LinkedIn
**S13**	AUS	2015	NFP	Australia Institute	Too close for comfort	Fossil fuels	Individual	None	Includes references. Right-to-information releases, including LinkedIn, news articles, and industry websites
**S14**	AUS	2018	Media	Michael West Media	Revolving Doors	Fossil fuels	Individual	Limited	Links included for individuals, e.g. for LinkedIn, personal webpages, Wikipedia etc.
**S15**	AUS	2018	NFP	Grattan Institute	Who’s in the room	Lobbying general; Revolving door general	Industry; Government	Limited	Australian Government Lobbyists Register, LinkedIn, Parlinfo.aph.gov.au, Wikipedia, news articles and various internet sources
**S16**	AUS	2017	Media	Sydney Morning Herald	Canberra Inc	Lobbying general	Company	None	None
**S17**	AUS	2018	Media	The Guardian	In the family’	Lobbying general	Industry; Government	Limited	Federal lobbyist register
**S18**	UK	2023	NFP	Campaign Against Arms Trade	Political Influence Browser	Military/Arms	Individual; Company	Comprehensive: extensive description of estimates, data selection and presentation, information sources	Comprehensive list of data sources included. Most data from FOI requests and official government transparency publications
**S19**	UK	2014 (updated 2018)	Government	Advisory Committee on Business Appointments (ACOBA)	Appointments taken up by former Crown servants (2014–2015 to 2017–2018)	Revolving door general	Individual	None	None
**S20**	UK	2017	NFP	Powerbase - Public Interest Investigations	Category: Revolving door	Arms; Finance; Revolving door general	Individual	None	References linked for each revolving door entry
**S21**	UK	2016	NFP/Media	Greenpeace (Unearthed)	Revealed: How the gas industry spent tens of millions of pounds lobbying UK & EU policymakers	Fossil fuels	Individual; Company	None	LinkedIn, trade accounts, FOI documents
**S22**	UK	2016	Media	Private Eye	Public servants, private paydays	Revolving door general	Individual	None	ACOBA records
**S23**	UK	2013	NFP	Global Justice Now (formerly World Development Movement)	Web of power	Fossil fuels; Finance	Individual; Company	None	Reference list included
**S24**	UK	2015	NFP	High Pay Centre	The revolving door	Revolving door general	Individual	None	ACOBA records
**S25**	UK	2015	NFP	Spinwatch - Public Interest Investigations	Access all areas	Fossil fuels; Lobbying general	Individual; Company	None	References linked in article. Uses data from fracking page on Powerbase.
**S26**	UK	2011	NFP	Spinwatch - Monitoring PR and Spin	Revolving door is unhealthy	Health	Individual	None	None
**S27**	UK	2023	NFP	Transparency International UK	Managing revolving door risks in Westminster	Revolving door general	Industry	None	Reference list included. Data from ACOBA letters and Campaign Against the Arms Trade were used
**S28**	USA	2024 (ongoing)	NFP	OpenSecrets	Revolving door overview	Revolving door general	Individual; Company; Industry; Government	Comprehensive: describes project intent, conceptualisation, operational definitions, data sources, industries represented	Core set of data from Columbia Books. Other data from internal resources and publicly available sources
**S29**	USA	2021	NFP	Tech Transparency Project (TTP)	Crypto Industry Amasses Washington Insiders	Crypto	Individual; Government	None	Lobbying disclosures, corporate filings, non-profit tax records, legal filings, LinkedIn profiles, press reports and other public records
**S30**	USA	2020	Academic	LobbyView	Lobbyist Data	Lobbying general; Revolving door general	Individual	Comprehensive: Description of data variables and system described for each data set, including a codebook pdf	US Senate Lobbying Disclosure Act Database
**S31**	USA	2016	Media	The Intercept	The Android administration	Tech	Company; Government	Limited	Publicly available information: LinkedIn profiles, news sources, lobby disclosure records, and OpenSecrets.org data
**S32**	USA	2015	NFP	Sunlight Foundation & OpenSecrets	All Cooled Off	Lobbying general	Individual; Government	Limited	House Clerk’s post-employment notifications
**S33**	USA	2013	NFP	Project on Government Oversight (POGO)	Dangerous Liaisons	Security/Fraud	Individual; Company; Government	Limited (links to external methods pages for POGOs SEC database did not work)	Interviews with current and former SEC officials, federal records obtained through FOI
**S34**	USA	2003	NFP	CorpWatch, Global Exchange, Public Citizen	Bechtel: Profiting from Destruction	Engineering; Construction	Individual; Company	None	Reference list included
**S35**	USA	2022	NFP	Wikipedia	Revolving door (politics) – United States	Revolving door general	Individual	None	References linked
**S36**	USA	2018	Academic	Bush School of Government and Public Service	Lobbying After Federal Service	Lobbying general; Revolving door general	Industry; Government	Comprehensive: description of database population, database variables, variable sources, data collection, data cleaning, data adjustment, data analysis, missing data	Plum Book, LinkedIn, Glassdoor, Federalpay, Guidestar, LDA database, OpenSecrets, Google, executive agencies’ websites
**S37**	USA	2018 (revised 2024)	NFP	National Bureau of Economic Research (NBER)	From revolving doors to regulatory capture?	Trademarking & patents	Company; Government	Some methodology regarding data sources, dataset construction, and summary statistics described throughout	Patent Examination Research Dataset; Patent Practitioner Rosters; Thomson Innovation; Educational histories from various websites and directories
**S38**	USA	2023	Government	Senator Elizabeth Warren	Pentagon Alchemy	Military/Arms	Company; Government	Limited	OpenSecrets Revolving Door database, corporate websites, lobbying disclosures, and U.S. Senate confirmation lists
**S39**	USA	2019	Government	Congressional Research Service	Executive Branch Service and the “Revolving Door” in Cabinet Departments	Lobbying general	Government	Comprehensive: describes data sources, inclusion criteria and linking steps	Plum Book, Lobbying Disclosure Act data
**S40**	USA	2021	Government	U.S. Government Accountability Office (GAO)	Post-government employment restrictions	Military/Arms	Company; Industry; Government	Comprehensive: describes scope and objectives, definitions, data sources, assessing reliability, data collection	DOD and Internal Revenue Service data. Interviews with contractors, ethics officials from DODs Standards of Conduct Office (SOCO), Defence Pricing and Contracting officials, Inspector General officials. SOCO website content. Surveys of major defence contractors
**S41**	USA	2014	NFP	U4 Anti-Corruption Resource Centre	The Revolving Door Indicator	Finance	Company	Limited	Company websites, LexisNexis Academic, OneSource (Avention), OpenSecrets.org website, government agency websites (SEC and Treasury), social network websites (LinkedIn), and business websites (Businessweek, Business Insider, Bloomberg).

Most records analysed the revolving door at multiple levels, with 34 (83%) looking at individuals, 19 (46%) at companies and 16 (39%) at the level of governments (these often occurred in the same record). Of the records analysing individuals, 31 named all or some individuals, while three did not name individuals (S17, S36, S40). 33 of the records also named specific companies and industry sectors linked to the revolving door. In most cases it was straightforward to quantify the units of analysis. However, in some cases, such as for larger databases like Open Secrets (S28) it was more challenging to identify the exact number of revolvers, as a variety of data sets were available that examined different aspects of the revolving door (e.g. Industries or Former Members of Congress). The number of total revolvers included in each dataset varied considerably from a minimum of 10 cases (S26, S34), to as many as 5820 (S30). Supplementary File 2 contains further details about the units of analysis.

Only 7 records (17%) provided detailed methodology descriptions (S18, S28, S30, S36, S39, S40, S41). Most records (34, 83%) provided no description of their methodology or only a basic description of which individuals or companies were studied or how data was collected. Nearly all resources except four (S1, S16, S19, S26) included a reference list or linked sources – meaning data sources were the most traceable part of the research process. Common data sources included lobbyist registers, disclosure records, LinkedIn, government or company websites, freedom of information requests, news outlets, and other public records. Only three records (S6, S33, S40) described more active primary data collection methods like interviews or surveys.

### How useable and reuseable are the datasets and resources

**Fig. 2:**
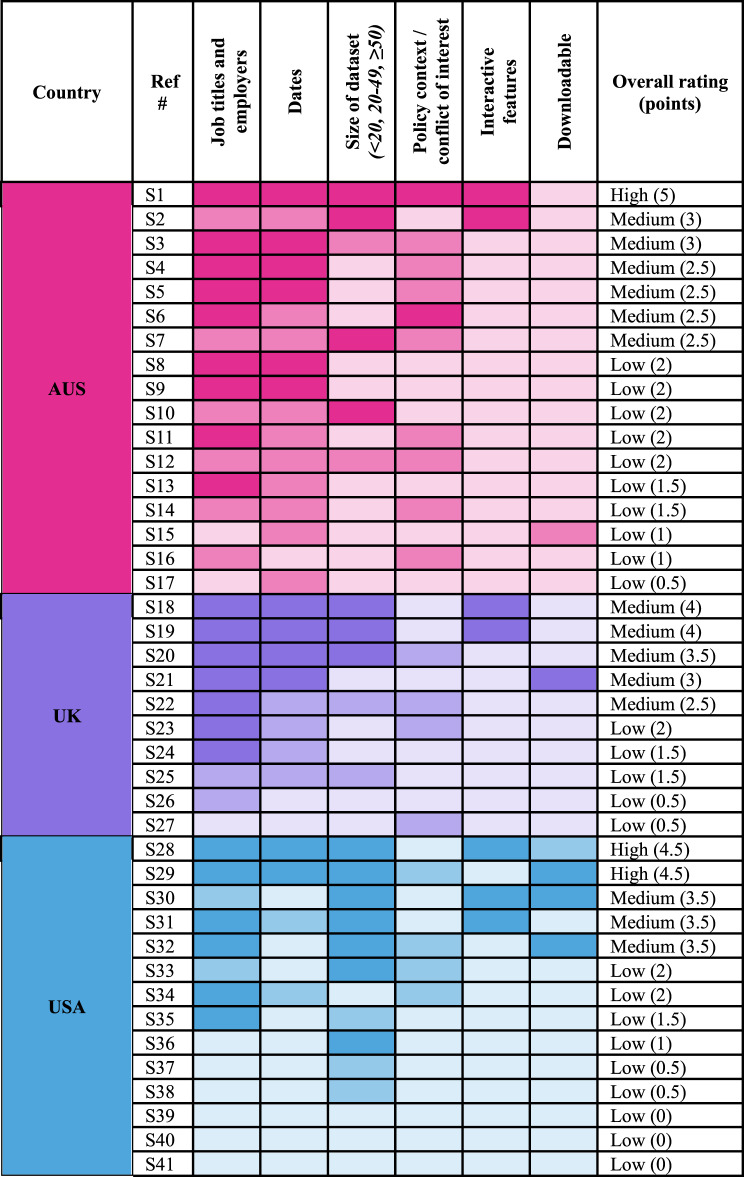
Heatmap measuring levels of data use and reuse (darker colours indicate a higher score)

Most records exhibited a low (24, 59%) or medium (14, 34%) level of data use or reuse. We developed a heatmap (Fig. 2) to provide a quick reference for researchers to identify best practice examples of data reusability (e.g. interactive features or downloadable data). Only three records (7%) demonstrated a high level of data use or reuse. These resources were typically large, interactive databases with detailed revolving door information. In Australia, Michael West Media’s database (S1) included a search engine, industry categories, filters by political party, and detailed bibliographies for each revolver that at times included policy context or potential conflicts of interest. In the USA, OpenSecrets (S28) provided one of the largest and richest datasets we encountered, with several different categories of data, including for industries, former members of Congress, lobbying firms and revolvers by administration. However, Open Secrets’ revolving door data could not be downloaded as it is under copyright. In contrast, the third high-ranking record from Tech Transparency Project (S29) offered a downloadable spreadsheet including names, dates, and job titles for 235 revolvers.

Across the six categories we evaluated, job titles and employers and dates of employment were the most frequently available types of information provided. In contrast, it was very rare to have downloadable or interactive data. Data could only be fully downloaded for four resources (S21, S29, S30, S32) or partially from two (S15, S28).

The quality and consistency of data varied considerably across the resources. For example, the Australian Centre for Public Integrity’s list had mostly unspecific roles and positions listed, such as ‘consultant’ or ‘director.’ The category regarding policy context or potential conflicts of interest presented the greatest variation in available data and the most boundary cases. A very small number of resources (S1, S6) provided rich details about potential conflicts of interest or policy context for most revolvers. Instead, more frequently this detailed information was only illustrated for a small number of cases. Most often, resources described the risk of influence or conflicts of interest in a very general sense, but did not link this to specific examples for individuals, particularly in reports where the revolving door only makes up one type of corporate influence examined.

A number of the records reflected on challenges in studying the revolving door. Common themes included a lack of transparency generally, unenforced regulations and limited scope of laws on disclosure, lobbying, cooling-off periods (see Supplementary File 2 for further detail). Other unique challenges were described in US records, such as an absence of indicators to actually measure distortions caused by the revolving door (S41) as well confusing post-employment laws with loopholes that allow former members of government to ‘advise’ private companies (S32).

## Discussion

Our study examined non-academic efforts to document and report the revolving door across three jurisdictions. Across the 41 records we analysed, we found common approaches to analysing the revolving door. Most focused on ‘revolvers’ (i.e. people who have a revolving door background) generally – as opposed to industry case studies. Most also focused on individual ‘revolvers’ as the main unit of analysis – as opposed to patterns across government departments or companies. While the relatively small number of records limits our capacity to generalise, we can observe that industry cases followed a pattern across jurisdictions, with Australian records focusing more on fossil fuels, in comparison to a focus on defence and technology in the United States. Considering the importance of these industries to each country’s respective economies, this emphasis is understandable. Finally, while the overall approach for each record was often similar, we found significant diversity in the data sources used and the organisation and presentation of data. Below, we reflect on some of the patterns and themes we observed in our study. We also consider how our study could inform future research of the revolving door and commercial determinants of health scholarship more generally.

While we identified a diversity of data sources used to analyse the revolving door across the records we analysed, many of these sources were restricted to specific county contexts. The Plum Book, for example, is a resource provided by the US government of current and past political appointees in the US federal government [19]. To the best of our knowledge, no such a consolidated resource is available in other contexts [8]. Our study suggests that baseline data available from governments largely dictate the breadth of revolving door reporting and have flow on effects for the amount of human resources needed to collect additional data. We can see this clearly in the US context, where government legislation requires disclosure of the revolving door in quarterly lobbying reports [24]. There are also government resources devoted to cleaning, reproducing and updating the data, making it readily useable for research purposes. Corporate political influence notwithstanding, the US is generally ranked as having the best transparency around lobbying (though off a very low base we note) [24]. US studies on lobbying and the revolving door are so prevalent precisely because data are so accessible. While a global audit of revolving door datasets has not yet occurred, other studies analysing the availability of lobbying data suggest that few countries provide robust and comprehensive data [8]. This highlights a particularly wicked problem for revolving door research (and corporate political activity research more generally) – namely that government data is often the most important data source, yet in many countries it is often poor quality or non-existent. To address this, we need both a long-term strategy to reform legislation and require better data sharing as well as efforts in the short term to find creative approaches to consolidate existing information about the revolving door and fill in the gaps where government data falls short.

Leveraging opportunities to consolidate revolving door data brings us to the importance of data use and reuse. We found that most records did not provide a high level of data use or reuse. Instead, they offered snapshots into specific individuals or sectors but lacked a systematic approach to data organisation. Many of the records in our study would be time-consuming to extract data from, severely limiting reusability. While many of the media articles were unlikely to be published with data reuse in mind, several of the other records were clearly designed to be used as a tool to understand the revolving door. From these, we have identified a few features that make it easier to use and reuse the data. Providing data in easily downloadable formats that are machine readable (e.g. a spreadsheet) eliminates much of the time-consuming labour of collecting, organising and classifying data. Classifying the data (e.g. assigning an industry sector to the employer or documenting the political party of a politician) likewise makes it easier to connect to other data sources (such as political donations or lobbying data). As a researcher, the reusability of data is important (and in the case of the revolving door, especially valuable considering the often-intensive human labour required to collect the data in the first place) [13]. A few of the records we analysed linked their revolving door data to other datasets, offering a glimpse of benefits of data linkage. Open Secrets and LobbyView both linked their revolving door data to their lobbyist datasets, allowing researchers to analyse, for example, how frequently a lobby firm or industry used revolving door lobbyists [51]. Likewise, the Campaign Against Arms Trade record linked their revolving door data with published departmental meetings data for senior government officials in the UK, albeit they note that meeting data has become more difficult to collect in recent years [52]. While data linkage is challenging, the potential insights it offers (such as comparing the political strategies of different industries) makes its pursuit extremely worthwhile [53].

In addition to exploring options for data linkages, deeper analysis of the revolving door can help respond to calls for commercial determinants of health (CDoH) researchers to diversify their disciplinary engagement [54, 55]. For example, quantitative analysis of the revolving door [12, 41] – especially at larger scales that considers the whole population of ‘revolvers’ – naturally intersects with political science scholarship. While the large-n studies of political science help to identify patterns (for instance, that revolving door lobbyists are more common in consultant firms [56]), the more industry-focused CDoH research can help to spot anomalies in the patterns and develop explanations for them. For example, a study of tobacco industry government relations personnel and lobbyists found almost half to have a revolving door background, when political science studies suggest that the in-house number should be much lower [12, 56]. This single artifact opens the door for a rich discussion of the potential impact of the tobacco industry’s reputation, Australia’s history of tobacco control and the current policy and regulatory context in explaining the deviation of the tobacco industry from the patterns seen in other industries.

Studies of revolving door also present opportunities for theory testing and development – something that has been flagged as a common lacuna in CDoH scholarship [55]. The causal mechanisms and process-tracing highlighted by Gomez, for instance, would be well-suited to exploring and explaining the potential influence of revolving door activities on the development of a particular policy over time relative to other corporate political activities. Analysis of the revolving door could also be used to advance theory development around state-business relationships. For example, recent efforts to conceptualise commercial actors in the CDoH discipline have highlighted the ‘blurriness’ between public and private sectors [57]. To date, state-business relationships have often been explored in a more structural sense, such as international relations scholars’ discussions of the ‘hollowing out of the state’ [58, 59]. Consideration of the importance of personal relationships as a form of authority and power could provide theoretical support to institutional analyses of how and why businesses are powerful. This could further complement work on conflicts of interest in policy making, extending, for example, Baum and colleague’s recent work analysing the rise of consultants and what we might conceptualise as the ‘thinning out’ of the public sector [60].

One challenge we observed in this study (and in our other work analysing corporate political influence) is the challenge of navigating intellectual property rules and ethics requirements of disciplines and universities. The Open Secrets dataset illustrates how IP rules can restrict opportunities to use and reuse data. Their revolving door database is based on an original dataset of lobbyist biographies that is commercially available for purchase – because of this they cannot allow their full dataset to be downloaded, even though they have expanded on the original dataset substantially [15]. Other datasets potentially relevant to the revolving door likewise have certain IP constraints around their use. One of the most common data sources used to analyse the revolving door is the professional network platform LinkedIn. LinkedIn is quasi-publicly available; however, it does not allow users to download large amounts of information at once (and it has pursued legal action against companies and individuals for scraping their data) [61]. Lastly, issues of individual rights to privacy compound existing challenges around data ownership and intellectual property. Revolving door research must navigate a delicate balance between individual privacy and the public’s right to knowledge. Revolving door data typically concerns individuals’ employment records – particularly for former government officials – information that is considered personal and potentially subject to privacy laws. Large language models, agentic AI and other advances in ‘big data’ have made publicly available data that was formerly ‘defacto private’ (because it was difficult to access) substantially more accessible. This has implications for principles and rules governing data privacy and data use [62]. Revolving door researchers (and CDoH researchers more broadly) will need to grapple carefully with ethical questions around how to balance individual privacy with public interest disclosures.

Our study had several limitations. We looked at only three, high-income countries. Future studies could widen the scope to explore approaches to understanding the revolving door in different country and political contexts. Our study was also limited to non-academic resources. In the interest of identifying different data sources and approaches to studying the revolving door, an audit of peer-reviewed studies of the revolving door would complement our work (though we note, as per academic ethics requirements, there are often – though not always [63] – restrictions on publishing the full dataset behind a research project) [12]. Our framework evaluating data use and reuse had several limitations. We only assessed six criteria (in the interest of study feasibility) and future studies could expand on these to interrogate a wider spectrum of features. In seeking to expand the ‘toolbox’ of corporate political influence researchers, we are interested to explore the breadth of potential attributes that could be collected concerning the revolving door to enrich future research projects. In particular, it is crucial for research to not only demonstrate the extent of the revolving door, but also its impacts on policy outcomes. A second limitation is that our framework took a simple approach to classifying different levels of data use and reuse, weighting all criteria the same, and only differentiating between three possible ratings. This meant that some resources with a single exceptional feature scored poorly overall, as they were missing other criteria. For example, Campaign Against Arms Trade record (S18) scored medium overall as it lacked policy context and was not downloadable (scoring zero for both categories). However, it had one of the most detailed employment timelines of any resource. Further, the tool was linked to data on government meetings so that it was possible to get a sense of the direct political influence of revolvers. In contrast, the ACOBA record (S19) scored the same as the CAAT record, however, its information is functionally less accessible and reusable, being organised in separate PDF files that require manual searching. Several of the resources that ranked poorly (e.g. S36–S41) did not have a focus on individuals, making the criteria of job titles or dates inapplicable for them. On the one hand, this highlights an opportunity for future studies to interrogate these datasets more closely or indeed reuse the data in them to conduct additional analyses. On the other hand, our findings speak to the need for governments and other data providers to move towards machine readable formats to make it easier to monitor the revolving door in the first place.

## Conclusion

It is our hope that this paper highlights both the importance of documenting and understanding the revolving door for public health advocates and some opportunities to do so. The absence of public health organisations in our findings indicates that the revolving door is simply not a priority for many public health advocates. On the one hand, the remit of public health is vast – corporate political activity is perhaps understandably ranked as a lower priority than pandemic preparedness or mental health or reproductive rights (amongst a myriad of options). Yet as commercial determinants of health scholars, we cannot help but see the political and business angle to every public health issue. Whether it is decisions over what drugs do or do not get government approval or the link between social media algorithms and mental health, public health is fundamentally shaped by business interests. To loosely paraphrase former WHO Director General Chan, the best and most equitable policies to protect and promote population health often threaten business interests [64]. Even if public health advocates do not make the revolving door or corporate political influence more generally a top priority, we suggest that they would do well to pay far more attention to the political machinations of businesses.

## Electronic supplementary material

Below is the link to the electronic supplementary material.

Supplementary Material 1

Supplementary Material 2

## Data Availability

The datasets analysed during the current study are available from the corresponding author on reasonable request.
