# Supplementary material for: Rethinking revolving door research: a scoping review of methods and datasets used by non-academics to examine the revolving door
Source: Global Health. 2026 Jan 16;22:22. doi: 10.1186/s12992-025-01184-7 (PMC12896196; doi:10.1186/s12992-025-01184-7)
Supplement: Supplementary file 1 — Supplementary Material 1 [file 12992_2025_1184_MOESM1_ESM.docx]

**Supplementary File 1**

1. **Conceptual categories and synonyms**

| **Conceptual category** | **Terms** |
| --- | --- |
| Revolving door | Revolving door |
| Commercial | “private sector” OR industry OR corporate OR commercial OR “private interest” OR “interest group” OR company OR industry OR business |
| Government | government OR politics OR “public service” OR “public office” OR “public sector” OR politician OR “public servants” OR public OR civil |
| Lobbying | lobbyist OR lobbying OR “lobby group” OR “lobby firm” OR “lobbyist register” |
| Conflict of interest | conflict of interest” OR risk OR influence OR “undue influence” OR “policy capture” OR “state capture” |
| Employment | job OR employment OR career OR profession |

**We also trialled other possible synonyms for the revolving door such as "job swapping" OR "post-government employment" OR "career change" OR "post-employment." However, these terms did not yield relevant results so were excluded from our search strategy.*

***For the government category, we trialled terms specific to different country contexts. For example, the terms parliament OR premier OR minister for the Australian context and congress OR president OR senator for the USA. However, these terms did not yield additional relevant results compared with our original keyword searches so were excluded from our search strategy.*

1. **Advanced Google search strategy**

We conducted three series of searches using Google’s regional filter to limit results to Australia, the UK and United States. Results were limited to the English language. The first 10 pages (100 results) were screened for each keyword search (700 results screened for each country).

| Country | Keyword search string # | Date and time | Number of hits | # results screened | # new records |
| --- | --- | --- | --- | --- | --- |
| AUS | “Revolving door” AND (government OR politics OR “public service” OR “public office” OR “public sector” OR politicians OR “public servants”) | 12.08.24, 09:40am | 52,500 | 100 | 26 |
| AUS | “Revolving door” AND (lobbyist OR lobbying OR “lobby group” OR “lobby firm” OR “lobbyist register”) | 12.08.24, 11:42am | 8,290 | 100 | 11 |
| AUS | “Revolving door” AND (“private sector” OR industry OR corporate OR commercial OR “private interest” OR “interest group”) | 12.08.24, 1:21pm | 52,800 | 100 | 5 |
| AUS | “Revolving door” AND (“conflict of interest” OR risk OR influence OR “undue influence” OR “policy capture” OR “state capture”) | 12.08.24, 3:16pm | 34,700 | 100 | 4 |
| AUS | Government AND private AND lobbyists AND influence | 13.08.24, 1:08pm | 27,300 | 100 | 2 |
| AUS | (government OR public OR civil OR politician) AND (company OR corporate OR industry OR business) AND (conflict OR influence OR capture OR lobby) | 17.02.25, 10:16am | 152,000,000 | 100 | 2 |
| AUS | (government OR public OR civil OR politician) AND (company OR corporate OR industry OR business) AND (conflict OR influence OR lobby) AND (job OR employment OR career OR profession) | 17.02.25, 12:21am | 13,100,000 | 100 | 2 |
| UK | “Revolving door” AND (government OR politics OR “public service” OR “public office” OR “public sector” OR politicians OR “public servants”) | 16.09.24, 09:24am | 46,000 | 100 | 8 |
| UK | “Revolving door” AND (lobbyist OR lobbying OR “lobby group” OR “lobby firm” OR “lobbyist register”) | 17.09.24, 11:34am | 10,100 | 100 | 2 |
| UK | “Revolving door” AND (“private sector” OR industry OR corporate OR commercial OR “private interest” OR “interest group”) | 17.09.24, 12:42am | 128,000 | 100 | 0 |
| UK | “Revolving door” AND (“conflict of interest” OR risk OR influence OR “undue influence” OR “policy capture” OR “state capture”) | 17.09.24, 1:56pm | 79,800 | 100 | 0 |
| UK | Government AND private AND lobbyists AND influence | 17.09.24, 3:20pm | 42,300 | 100 | 0 |
| UK | (government OR public OR civil OR politician) AND (company OR corporate OR industry OR business) AND (conflict OR influence OR capture OR lobby) | 26.02.25, 09:43am | 3,150,000,000 | 100 | 0 |
| UK | (government OR public OR civil OR politician) AND (company OR corporate OR industry OR business) AND (conflict OR influence OR lobby) AND (job OR employment OR career OR profession) | 26.02.25, 11:06am | 45,900,000 | 100 | 0 |
| USA | “Revolving door” AND (government OR politics OR “public service” OR “public office” OR “public sector” OR politicians OR “public servants”) | 29.08.24, 4:27pm | 1,300,000 | 100 | 6 |
| USA | “Revolving door” AND (lobbyist OR lobbying OR “lobby group” OR “lobby firm” OR “lobbyist register”) | 31.08.24, 12:21pm | 21,500,000 | 100 | 5 |
| USA | “Revolving door” AND (“private sector” OR industry OR corporate OR commercial OR “private interest” OR “interest group”) | 31.08.24, 3:37pm | 1,330,000 | 100 | 2 |
| USA | “Revolving door” AND (“conflict of interest” OR risk OR influence OR “undue influence” OR “policy capture” OR “state capture”) | 05.09.24, 10:41am | 765,000 | 100 | 5 |
| USA | Government AND private AND lobbyists AND influence | 05.09.24, 1:16pm | 3,090,000 | 100 | 0 |
| USA | (government OR public OR civil OR politician) AND (company OR corporate OR industry OR business) AND (conflict OR influence OR capture OR lobby) | 26.02.25, 2:18pm | 2,840,000,000 | 100 | 0 |
| USA | (government OR public OR civil OR politician) AND (company OR corporate OR industry OR business) AND (conflict OR influence OR lobby) AND (job OR employment OR career OR profession) | 26.02.25, 12:25pm | 1,420,000,000 | 100 | 1 |

1. **Targeted website searches**

*An initial list of organisations was sourced from the supplementary material of the papers: Aiding empirical research of the commercial determinants of health (Lacy-Nichols et al. 2023) and Mechanisms for addressing and managing the influence of corporations on public health policy, research and practice (Mialon et al. 2020). This list was supplemented by the corresponding authors’ knowledge of the topic. Each of the websites (if available in English) was searched for the term ‘revolving door to identify whether the organisation published research (e.g. reports) on the topic. For websites with a search function, we searched for the term ‘revolving door’ and for those without a search function, we reviewed their published reports for relevant content.*

| **Country or Region** | **Name of organisation** | **Date of search** | **# search results** | **# results preliminarily**  **screened** | **# results full-text screened** | **# included** |
| --- | --- | --- | --- | --- | --- | --- |
| Australia | Australia Institute | 18.09.24, 12:45pm | 41 | 41 | 2 | 1 |
| Australia | Australian Democracy Network (Our Democracy Campaign) | 18.09.24, 3:35pm | 0 | 17 | 0 | 0 |
| Australia | Australasian Centre for Corporate Responsibility (ACCR) | 19.09.24, 10:21am | 5 | 5 | 0 | 0 |
| Australia | Centre for Public Integrity | 19.09.24, 10:26am | 0 | 6 | 1 | 1 |
| Australia | Foundation for Alcohol Research and Education (FARE) | 19.09.24, 10:35am | 4 | 4 | 0 | 0 |
| Australia | Grattan Institute | 19.09.24, 10:39am | 6 | 6 | 0 | 0 |
| Australia | Human Rights Law Centre | 19.09.24, 10:47am | 0 | 1 | 0 | 0 |
| Australia | Pearls and Irritations | 19.09.24, 11:11am | 116 | 116 | 2 | 1 |
| Australia | Transparency International Australia | 19.09.24, 11:45am | 34 | 34 | 1 | 0 |
| Australia | 350 Australia | 19.09.24, 11:58am | 0 | 10 | 1 | 0 |
| UK | Spotlight on Corruption | 19.09.24, 2:58pm | 13 | 13 | 0 | 0 |
| UK | Tobacco Control Research Group | 19.09.24, 3:20pm | 2 | 2 | 0 | 0 |
| UK | Transparency International UK | 22.09.24, 09:49am | 57 | 57 | 0 | 0 |
| UK | Unlock Democracy | 19.09.24, 2:05pm | 4 | 4 | 0 | 0 |
| UK/Europe | Spinwatch | 19.09.24, 2:09pm | 50 | 50 | 3 | 3 |
| UK/International | openDemocracy | 22.09.24, 3:26pm | 339 | 339 | 6 | 2 |
| USA | Alcohol Justice | 20.09.24, 1:59pm | 0 | 6 | 0 | 0 |
| USA | Center for Media and Democracy (CMD) | 20.09.24, 2:06pm | 4 | 4 | 0 | 0 |
| USA | Center for Responsive Politics | 20.09.24, 2:19pm | 4 | 4 | 0 | 0 |
| USA | Center for Science in the Public Interest (CSPI) | 20.09.24, 2:23pm | 0 | 0 | 0 | 0 |
| USA | CounterCorp | Website not available | - | - | - | - |
| USA | Corporate Research Project | 21.09.24, 12:53pm | 9 | 9 | 0 | 0 |
| USA | Corporate Watch | 20.09.24, 2:25pm | 24 | 24 | 2 | 0 |
| USA | Corpwatch | 21.09.24, 1:20pm | 52 | 52 | 1 | 1 |
| USA | U.S. Right to know (URTK) | 21.09.24, 1:02pm | 4 | 4 | 0 | 0 |
| USA | LobbyView | 12.03.25, 10:21am | 0 | 7 | 7 | 1 |

1. **Results excluded during full text screening**

| **AUS excluded records from Google searches** | | | | |
| --- | --- | --- | --- | --- |
| **#** | **Organisation** | **Year** | **Title** | **Reason for exclusion** |
| 1 | Mandarin | 2021 | Opinion: The triumph of the lobbies - Australia's revolving door and democracy | Data not organised or formatted |
| 2 | The Age | 2021 | ‘Revolving door’ between public service and consultancies | Insufficient units of analysis |
| 3 | Michael West Media Independent Journalists | 2020 | Brothers-in-Arms: the high-rotation revolving door between the Australian government and arms merchants | Insufficient units of analysis |
| 4 | ABC | 2023 | The path from MP to big business is well trodden — and defence and AUKUS are in their sights | Insufficient units of analysis |
| 5 | Sydney Morning Herald | 2023 | Corporations exploiting ‘weak integrity laws’ to influence government: Report | Duplicated data from included record (Centre for Public Integrity 2023 report) |
| 6 | Parliament of Australia | 2019 | Government Senators' Dissenting Report | Insufficient units of analysis |
| 7 | Accounting Times | 2024 | Big 4 ‘revolving door’ drawing ire around the world | Insufficient units of analysis |
| 8 | Grattan Institute | 2019 | Submission to the Senate’s ‘revolving door’ inquiry | Duplicated data from included record (Grattan Institute 2018 report) |
| 9 | Sydney Morning Herald | 2019 | Tighten the rules on the revolving door of politician lobbyist | Insufficient units of analysis |
| 10 | Australia Institute | 2024 | Removing the fossil fuel industry’s influence on politics and parliament is how we change the story and restore trust in government decisions. | Insufficient units of analysis |
| 11 | GreenLeft | 2024 | From PM to arms industry: Scott Morrison joins 'revolving door' trend | Insufficient units of analysis |
| 12 | Independent Australia | 2020 | The revolving door inside our aged care sector | Insufficient units of analysis |
| 13 | Accountability Round Table | 2019 | Lobbyists | Insufficient units of analysis |
| 14 | Australian Conservation Foundation | 2015 | Connections between Australian politics and coal lobby run deep | Insufficient units of analysis |
| 15 | Centre for Public Integrity (commissioned LockTheGate) | 2024 | In whose interest? The case for reforming the Northern Territory lobbying regime | Other focus (Advisory Commission, not strictly revolving door) |
| 16 | Echo | 2023 | International revolving doors | Data not organised or formatted |
| 17 | Human Rights Law Centre | 2023 | Selling out: how powerful industries corrupt our democracy | Insufficient units of analysis |
| 18 | Declassified Australia | 2023 | Sinking billions - revolving doors (Part 2) | Other focus (Expert advisory panel, not strictly revolving door) |
| 19 | ABC News | 2012 | Get to know your lobby groups | Data not organised or formatted |
| 20 | The Mandarin | 2019 | Australia’s political lobbying regime is broken and needs urgent reform | Insufficient units of analysis |
| 21 | Independent Australia | 2021 | Frydenberg's revolving door | Insufficient units of analysis |
| 22 | Financial Review | 2009 | The trail of lobbyists leads to Bligh's office | Data not organised or formatted |
| 23 | Independent Australia | 2019 | Christopher Pyne and the Canberra revolving door | Insufficient units of analysis |
| 24 | Get Up | 2021 | Ben Wyatt waltzes through the revolving door between government and Big Mining | Insufficient units of analysis |
| 25 | Legislative Council Legal and Social Issues Committee | 2021 | Following the money - the privatisation of Australian prisons (Attachment for inquiry into Victoria's criminal justice system submission) | Unable to access full text (redacted sections) |
| 26 | Renew Economy | 2023 | Revolving doors, golden escalators and the demise of climate and energy policy | Duplicated data from included record (Grattan Institute 2018 report) |
| 27 | Sydney Morning Herald | 2023 | Orange is the new hack: 891 more lobbyists gain inside access to Parliament House | Other focus (sponsored passes for lobbyists to access government) |
| 28 | The Conversation | 2016 | The revolving door: why politicians become lobbyists, and lobbyists become politician | Insufficient units of analysis |
| 29 | Australasian Centre for Corporate Responsibility | 2021 | Gaslighting: How APPEA and its members continue to oppose genuine climate action | Insufficient units of analysis |
| 30 | The Mandarin (Premium) | 2019 | Deep links between the private sector and Defence have been to the nation’s benefit. But it’s complicated. The department has expanded its toolbox to manage this ‘revolving door’ | Unable to access full text (requires subscription) |
| 31 | Crikey | 2014 | Martin Ferguson’s revolving door puts energy industry in a spin | Insufficient units of analysis |
| 32 | Undue Influence | 2023 | Mapping the revolving door between government and the weapons industry: Undue Influence revolving door database progresses | Insufficient units of analysis (Database that does not exist yet) |
| 33 | Financial Review | 2023 | Big four hiring ex-MPs, department heads a ‘conflict-of-interest risk’ | Data not organised or formatted |
| 34 | Australia Democracy Network | 2022 | Confronting State Capture | Data not organised or formatted |
| 35 | Open Politics | 2024 | Open Politics: Private interests of Australia's politicians | Other focus (private interests of Australian politicians) |
| 36 | Sydney Morning Herald | 2024 | How big business’ lobby groups are using you to pressure the government | Insufficient units of analysis |
| 37 | The Australian | 2022 | Key Andrews ministerial aide joins lobby firm | Insufficient units of analysis |
| 38 | Australia Institute | 2023 | Consultants: corrosive and conflicted - Submission to NSW inquiry into the Government’s use of consultants | Other focus (consultancy work) |
|  | | | | |
| **UK excluded records from Google searches** | | | | |
| **#** | **Organisation** | **Year** | **Title** | **Reason for exclusion** |
| 1 | The Ferret | 2024 | Revealed: The revolving door between fossil fuel industry and the UK Government | Unable to access full text (requires subscription) |
| 2 | The Ferret | 2019 | Revealed: the ‘revolving door’ between politics and lobbying in Scotland | Unable to access full text (requires subscription) |
| 3 | BBC | 2021 | Which MPs have second jobs and how much do they earn? | Other focus (second jobs) |
| 4 | Transparency International UK | 2015 | Lifting the lid on lobbying: the hidden exercise of power and influence in the UK | Insufficient units of analysis |
| 5 | Guardian | 2024 | Six top politicians who exited Westminster via the ‘revolving door’ | Insufficient units of analysis |
|  | | | | |
| **USA excluded records from Google searches** | | | | |
| **#** | **Organisation** | **Year** | **Title** | **Reason for exclusion** |
| 1 | Center for Economic and Policy Research |  | The Revolving Door Project: Personnel Map | Other focus (economic sector’s interests in non-career positions) |
| 2 | Project on Government Oversight | 2021-2023 | Pentagon Revolving Door Database | Unable to access full text (database retired) |
| 3 | LegiStorm | 2024 | Unlock Revolving Door Tracking with a LegiStorm subscription | Unable to access full text (requires subscription) |
| 4 | Seattle Times & Northwest News Network | 2020 | From elected official to lobbyist in just days: Revolving door raises questions about powerful influences in Olympia | Unable to access full text (requires subscription) |
| 5 | CEPR | 2014 | The revolving door and worker flows in banking regulation | Insufficient units of analysis |
| 6 | Transparency International | 2014 | Grand Political Corruption in Democracies: Features, Programming Options and Assessment Tools | Insufficient units of analysis |
| 7 | State of California - Fair Political Practices Commission |  | Second Quarter Update | Insufficient units of analysis |
|  | | | | |
| **Excluded records from targeted website searches** | | | | |
| 1 | Australia Institute | 2017 | Undermining our democracy: Foreign corporate influence through the Australian mining lobby | Insufficient units of analysis |
| 2 | Pearls and Irritations | 2023 | Sinking Billions – Revolving Doors – Part 2 | Other focus (Committee, not strictly revolving door) |
| 3 | Transparency International Australia | 2023 | Picking a winner: How the gambling sector uses lobbyists to influence Canberra | Insufficient units of analysis |
| 4 | 350Australia | 2021 | Fossil Fuel Watch | Other focus (Commission, not strictly revolving door) |
| 5 | Open Democracy | 2021 | ‘Revolving door’ watchdog has met only once since Johnson’s election win | Insufficient units of analysis |
| 6 | Open Democracy | 2018 | Other revolving doors in the US | Insufficient units of analysis |
| 7 | Open Democracy | 2022 | Fears ex-BP and British Gas bosses could ‘sway’ government energy policy | Insufficient units of analysis |
| 8 | Open Democracy | 2013 | Milburn, the NHS, and Britain's 'revolving door' | Insufficient units of analysis |
| 9 | Corporate Watch | 2022 | Heat the rich? Part six: British gas/Centrica | Insufficient units of analysis |
| 10 | Corporate Watch | 2021 | Vaccine capitalism: five ways Big Pharma makes so much money | Insufficient units of analysis |
| 11 | LobbyView | 2024 | Datasets – Report Level Data | Other focus (lobbying report filings) |
| 12 | LobbyView | 2024 | Datasets – Client Level Data | Other focus (clients) |
| 13 | LobbyView | 2024 | Datasets – Bill Level Data | Other focus (bills) |
| 14 | LobbyView | 2024 | Datasets – Issue Level Data | Other focus (issues lobbied) |
| 15 | LobbyView | 2024 | Datasets – Network Data | Other focus (networks) |
| 16 | LobbyView | 2024 | Datasets – Text Data | Other focus (descriptions of lobbyist activities) |

1. **Heatmap criteria and scoring**

|  | **Downloadable** | **Interactive** | **Dates** | **Job titles** | **Context** | **Size** |
| --- | --- | --- | --- | --- | --- | --- |
|  | *Link to external Excel spreadsheet or other document* | *Moveable timelines or graphs, filters, search tools* | *Public and private sector dates of employment* | *Public and private sector job titles* | *Policy context or involvement, possible conflicts of interest (COI)* | *Number of data entries* |
| YES  1pt | All revolving door data can be downloaded | Interactive feature(s) included | Start and end dates for both public and private sector roles included | Specific job title and employer for public and private sector roles are included | References to potential COI or policy context available for most data entries | Dataset comprises of 50 or more data entries |
| PARTIAL OR INCOMPLETE  0.5pts | A portion of the data is available to download | (n/a) | Only partial or incomplete dates provided (e.g. only cessation date) | Only partial or insufficient job title information is provided (e.g. job titles only included for some roles), or positions are non-specific (e.g. ‘employed by a minister’) so that a clear position remains unclear | Policy context or possible COI only included for small number of data entries (e.g. case study examples) | Dataset comprises of between 20 and 49 data entries |
| NO  0pts | None of the data is available to download | No available interactive features | Employment dates not included | Job titles/positions are not described | No reference to potential COI or policy context. Or if mentioned, only described at a general level without providing examples linked to individuals | Dataset comprises of less than 20 data entries |

| **Scoring (across all criteria)** |
| --- |
| Low level: 0 – 2 points |
| Medium level: 2.5 – 4 points |
| High level: 4.5 – 6 points |
