# Supplementary material for: Rethinking revolving door research: a scoping review of methods and datasets used by non-academics to examine the revolving door
Source: Global Health. 2026 Jan 16;22:22. doi: 10.1186/s12992-025-01184-7 (PMC12896196; doi:10.1186/s12992-025-01184-7)
Supplement: Supplementary file 2 — Supplementary Material 2 [file 12992_2025_1184_MOESM2_ESM.docx]

**Supplementary File 2**

1. **Details of organisation funding and purpose**

| **Ref #** | **Country** | **Organisation** | **Type** | **About/Purpose** | **Affiliations & Funding** | **Website** |
| --- | --- | --- | --- | --- | --- | --- |
| S1; S14 | AUS | Michael West Media | Independent media publisher. Unclear if NFP or for-profit. | “Michael West Media is an independent media publisher covering the rising power of corporations over democracy. Our investigations focus on big business, particularly multinational tax-avoiders, financial markets and the banking and energy sectors.”  <https://michaelwest.com.au/about/> | “We are non-partisan, do not take advertising and are funded by readers.”  <https://michaelwest.com.au/about/> | <https://michaelwest.com.au> |
| S2; S9 | AUS | Centre for Public Integrity | NFP independent think tank | “The Centre’s goal is to bolster Australia’s democratic foundations and build its resilience. Our method for achieving this is to convene Australia’s leading integrity experts, identify key failings, and utilise Australia’s leading integrity experts to help produce evidence-based policy solutions. The board sets the strategic research priorities.”<https://publicintegrity.org.au/what-we-stand-for/> | “The Centre for Public Integrity is a registered Research Institute funded by generous donations from philanthropic foundations and the public. We proudly maintain our independence by refusing donations from political parties, unions, religious organisations, or entities whose values do not align with our mission.”  Major donors for 2024 include: McKinnon Foundation, Myer Foundation, Mannifera, Australia Communities Foundation  <https://publicintegrity.org.au/what-we-stand-for/> | <https://publicintegrity.org.au> |
| S3; S10; S11 | AUS | Crikey | Private for-profit digital media outlet | “Crikey is an independent Australian source for news, investigations, analysis and opinion focusing on politics, media, economics, health, international affairs, the climate, business, society and culture.”  <https://www.crikey.com.au/about/> | “As a legal entity, *Crikey* is a wholly owned subsidiary of Private Media Pty Ltd. Private Media is a privately owned company. Eric Beecher is the largest shareholder with slightly more than 40% of the company. Eric is also the chairman of the company.  Other significant shareholders include the Fairfax family via their family office Marinya Capital, and Cameron O’Reilly via Bayard Capital. Notable smaller shareholders include Paul Barry and Allen and Unwin Pty Ltd. None of these shareholders have any involvement in the day to day running of the company, including our editorial work.”  <https://www.crikey.com.au/2024/10/16/who-owns-crikey/> | <https://www.crikey.com.au> |
| S4; S13 | AUS | Australia Institute | Independent think tank, registered NFP | “The Australia Institute provides intellectual and policy leadership. We design work and research to influence and persuade policymakers, politicians and to shift the public debate. We are fiercely non-partisan but we don’t shy away from engaging in politics and political debates. We believe in democracy and our work is aimed at a better, more informed democratic debate.”  <https://australiainstitute.org.au/about/> | “Funded by donations from philanthropic trusts and individuals, and grants and commissioned research from business, unions, and non-government organisations”  <https://australiainstitute.org.au/about/> | <https://australiainstitute.org.au> |
| S4; S5 | AUS | Australian Conservation Foundation | NFP membership-based environmental NGO | “We’re the Australian Conservation Foundation, Australia's national environment organisation. We influence governments and businesses to protect the animals, rivers and reefs close to our hearts and hold decision-makers to account without fear or favour. Everything we do is evidence-based and helps nature and people thrive for generations to come.”  <https://www.acf.org.au/about> | “Our community’s incredible support means ACF is proudly independent, non-partisan and funded by donations.”  <https://www.acf.org.au/about> | <https://www.acf.org.au> |
| S5 | AUS | Australian Democracy Network | Registered NFP entity | “The Australian Democracy Network is creating a healthy Australian democracy that puts people and the planet first. We bring people and organisations together to campaign for the changes that make our democracy more fair, open, participatory, and accountable.”  <https://australiandemocracy.org.au/about> | Funders listed on website: Mannifera, Australian Communities Foundation; Rio Industrial Group, Reichstein Foundation, The Coalition, Paddy Pallin Foundation, Myer Foundation, Morris Family Foundation, Tripple  <https://australiandemocracy.org.au/about> | <https://australiandemocracy.org.au> |
| S5 | AUS | Human Rights Law Centre | NFP NGO providing legal advocacy for human rights | “We work in partnership with people and communities to advance human rights.We use strategic legal action and advocacy to defend hard-won human rights progress.” <https://www.hrlc.org.au/who-we-are> | Majority of revenue through goods and services provided (83%). Other funding from donations (10%).  <https://www.acnc.gov.au/charity/charities/2467ce7b-39af-e811-a963-000d3ad244fd/profile>  Audited Financial Reports available  <https://www.hrlc.org.au/annual-reports/> | <https://www.hrlc.org.au> |
| S6; S21 | AUS; UK | Greenpeace | NFP NGO environmental organisation | “Greenpeace is an independent campaigning organisation that uses non-violent direct action to expose global environmental problems and to force solutions which are essential to a green and peaceful future.”  <https://www.greenpeace.org.au/about-us/> | “Our campaigns are 100% independently funded by supporters just like you so we can remain fiercely independent. We don’t accept funding from corporations, political parties or governments and remain free from their bias.”  <https://www.greenpeace.org.au/about-us/funding/>  Annual reports and financial statements available | <https://www.greenpeace.org.au> |
| S7 | AUS | The Conversation | NFP media organisation | “The Conversation Australia and New Zealand is a unique collaboration between academics and journalists, and is the world’s leading publisher of research-based news and analysis.”  <https://theconversation.com/au/who-we-are> | “Funding comes from partners from the university and research sector, some philanthropic organisations and more than 22,000 individual donors”.  <https://theconversation.com/au/who-we-are>  2024 Stakeholder report: 51% from donations by readers and trusts, 39% by universities and research partners, 10% from other services.  <https://cdn.theconversation.com/static_files/files/3573/2024_Stakeholder_Report_AU_NZ__%2810%29.pdf?1734410202> | <https://theconversation.com/au> |
| S8 | AUS | The Klaxon | Independent news outlet. Unclear if for-profit or NFP. | “The Klaxon is a completely independent Australian news outlet delivering top-quality news for our community. We shine a light on the systemic and structural improprieties currently flourishing within the collective blindspots of much of the general media.”  <https://theklaxon.com.au/about/> | “The Klaxon is 100% owned and operated by multi-award winning investigative journalist Anthony Klan”  <https://theklaxon.com.au/about/>  Funding model of newspaper unclear. | <https://theklaxon.com.au> |
| S10 | AUS | The Mandarin | Online news outlet, part of for-profit media company | “The Mandarin is dedicated to the public sector through our daily news coverage, sold-out events, careers portal and more”  <https://www.themandarin.com.au/about/> | Owned by Private Media. “Private Media is a privately owned company. Eric Beecher is the largest shareholder with slightly more than 40% of the company. Eric is also the chairman of the company. Other significant shareholders include the Fairfax family via their family office Marinya Capital, and Cameron O’Reilly via Bayard Capital. Notable smaller shareholders include Paul Barry and Allen and Unwin Pty Ltd. None of these shareholders have any involvement in the day to day running of the company, including our editorial work.”  <https://www.crikey.com.au/2024/10/16/who-owns-crikey/>  Funding model of The Mandarin unclear. At least partly funded by subscriptions (significant portion of content paywalled). | <https://www.themandarin.com.au> |
| S12 | AUS | 350 Perth (part of 350 Australia) | Local chapter of global climate-action NGO. Likely NFP. | “We are growing a grassroots movement to end fossil fuels and create community-led solutions to the climate crisis.” | Local group for 350Australia. “Our movement is funded by grants and donations — we don’t accept money from governments or fossil fuel corporations so that we can remain independent.”  <https://chuffed.org/project/350australia>  Financial overview available at ACNC  <https://www.acnc.gov.au/charity/charities/08ae4fe1-39af-e811-a960-000d3ad24282/profile> | <https://350.org.au> |
| S15 | AUS | Grattan Institute | NFP independent public policy think tank | “Grattan Institute produces high-quality public policy recommendations for Australia’s future. Since we were established in 2008, our independent research has helped shape the Australian policy conversation., from childcare to the COVID-19 response.”  <https://grattan.edu.au/about/> | List of endowment supporters - at least $1 million over institute's lifetime (Aus government, Vic government, UniMelb, BHP, Myer Foundation, NAB, Scanlon Foundation, Susan McKinnon Foundation, Summer Foundation). Also discloses all affiliate partners, senior affiliates, and affiliates outlined.  <https://grattan.edu.au/about/supporters/> | <https://grattan.edu.au> |
| S16 | AUS | Sydney Morning Herald | Newspaper, part of for-profit larger media group. | “Breaking news from Sydney, Australia and the world. Features the latest business, sport, entertainment, travel, lifestyle, and technology news.”  <https://www.smh.com.au> | Owned by Nine Entertainment (which merged with Fairfax Media in 2018) and is a publicly listed company.  Billionaire Bruce Gordon is the biggest shareholder, with a 25.1% stake in Nine through his private investment firm Birketu, and equity swaps through  Macquarie.. “Pendal is Nine’s largest shareholder behind Birketu and Macquarie, holding about 7.4 per cent. Cooper Investors holds a little over 5 per cent, as does State Street and Vanguard.”  <https://www.afr.com/companies/media-and-marketing/bruce-gordon-95-bulks-up-interest-in-nine-20240607-p5jk6t>  Funding model of the Sydney Morning Herald unclear. At least partly funded by subscriptions and advertising.  <https://www.nineforbrands.com.au/media-kits/sydney-morning-herald/> | <https://www.smh.com.au> |
| S17 | AUS | The Guardian (Australia) | Private media organisation | “Latest news, breaking news and current affairs coverage from across Australia from theguardian.com”  <https://www.theguardian.com/australia-news> | Ownership structure is made up of three key organisations:   - **“The Scott Trust**, the sole shareholder of Guardian Media Group. The purpose — and privilege — of the Trust is to secure the financial and editorial independence of the Guardian in perpetuity. - **The Scott Trust Endowment**, a fund built up following the prudent sale of assets with the core purpose of investing to provide financial returns to support Guardian journalism in perpetuity. - **Guardian Media Group**, the owner of Guardian News & Media (GNM) and publisher of the Guardian newspaper and website.”   <https://www.theguardian.com/about>  The newspaper itself includes “different kinds of paid content (One of four labels will appear on this content: ‘Supported by’, ‘Advertising partner/Exclusive advertising partner’, ‘Paid content/Paid for by’, or ‘Advertiser content/from our advertisers’)”  <https://www.theguardian.com/info/2016/jan/25/content-funding> | <https://www.theguardian.com/au> |
| S18 | UK | Campaign Against Arms Trade | NFP NGO (“private limited company by guarantee without share capital” | “Campaign Against Arms Trade (CAAT) is a UK-based organisation working to end the international arms trade.”  CAAT’s priorities are:   - To stop the procurement or export of arms where they might: exacerbate conflict, support aggression, or increase tension support an oppressive regime or undermine democracy, or threaten social welfare through the level of military spending. - To end all government political and financial support for arms exports - To promote progressive demilitarisation within arms-producing countries.   <https://caat.org.uk/about> | States “most funding from individuals”, details unclear  <https://caat.org.uk> | <https://caat.org.uk> |
| S19 | UK | Advisory Committee on Business Appointments (ACOBA) | Non-departmental government advisory body | ACOBA is no longer in operation (as of October 2025).  “ACOBA provided independent advice to former Ministers and the most senior servants on appointments they wish to take up on leaving government.”  <https://www.gov.uk/government/organisations/advisory-committee-on-business-appointments> | “The Committee is a non-statutory, non-departmental public body sponsored by the Cabinet Office”  <https://publications.parliament.uk/pa/cm201617/cmselect/cmpubadm/252/25205.htm> | <https://www.gov.uk/government/organisations/advisory-committee-on-business-appointments> |
| S20; S25; S26 | UK | Public Interest Investigations | NFP investigative journalism organisation | “Founded in 2005**, Public Interest Investigations (PII)** carries out cutting-edge research into key social, political, environmental and health issues in the UK and Europe.  Our work is underpinned by two key projects - this site, **Spinwatch.org** and a research wiki Powerbase.info, which focuses on the networks of powerful individuals and institutions shaping the public agenda.”  <https://spinwatch.org/index.php/about/about-spinwatch> | “**Spinwatch is a project of Public Interest Investigations, a non-profit company incorporated in England and Wales. It is run by an editorial collective of journalists and academics with decades of experience in the policy fields it covers.”**  <https://spinwatch.org/index.php/about/who-we-are>  “Spinwatch is funded by donations, royalties and proceeds from book sales and other writing and research commissions as well as by grant funding.”  <https://www.spinwatch.org.uk/about-spinwatch-mainmenu-13/3705-who-funds-spinwatch/> | <https://spinwatch.org> |
| S22 | UK | Private Eye | Satirical and investigative magazine Privately owned for-profit. | “Private Eye is the UK's number one best-selling news and current affairs magazine, offering a unique blend of humour, social and political observations and investigative journalism”  <https://www.private-eye.co.uk/about> | Privately owned by a group of individuals, and edited by Ian Hislop.  Funding model unclear. At least partly funded by subscriptions and advertising.  <https://www.private-eye.co.uk/about> | <https://www.private-eye.co.uk/about> |
| S23 | UK | Global Justice Now (formerly World Development Movement) | NFP NGO for global justice | “Global Justice Now works as part of a global movement to challenge the powerful and create a more just and equal world. We mobilise people in the UK for change, and act in solidarity with those fighting injustice, particularly in the global south.”  <https://www.globaljustice.org.uk> | Funded by Global Justice Now Trust - majority of income from donations (57%) and legacies, and about 30% from restricted grants.  <https://www.globaljustice.org.uk/about-us/how-were-funded-2/> | <https://www.globaljustice.org.uk> |
| S24 | UK | High Pay Centre | NFP think tank focused on pay and corporate governance | “The High Pay Centre is an independent, non-partisan think tank focused on the causes and consequences of economic inequality, with a particular interest in top pay. We run a programme of research, events and policy analysis involving business, trade unions, investors and civil society focused on achieving an approach to pay practices that enjoys the confidence of all stakeholders.” <https://highpaycentre.org/about/> | 2024 funding from: abrdn Financial Fairness Trust, Church of Engand Pensions Board, Trust for London.  2023 funding from: abrdn Financial Fairness Trust, Barrow Cadbury Trust, Chartered Institute of Personnel and Development, Trust for London  <https://highpaycentre.org/about/> | <https://highpaycentre.org> |
| S27 | UK | Transparency International UK | UK branch of global NFP NGO fighting corruption | “Transparency International UK is the UK chapter of Transparency International - a global movement sharing one vision: a world in which government, politics, business, civil society and the daily lives of people are free of corruption.”  <https://www.transparency.org.uk/who-we-are> | “We receive funding from a range of donors, including government agencies, multilateral institutions, foundations, the private sector and individuals. Funding may be unrestricted or tied to specific projects or programmes.” For 2024, 47% from government agencies, 21% multilateral organisations, 22% foundations and trusts.  <https://www.transparency.org/en/the-organisation/who-supports-us> | <https://www.transparency.org.uk> |
| S28; S32 | USA | Open Secrets (Centre for Responsive Politics) | NFP NGO research and transparency organisation | “OpenSecrets is the nation's premier research and government transparency group tracking money in politics and its effect on elections and policy.”  <https://www.opensecrets.org> | “OpenSecrets has operated since 1983 thanks to a combination of grants from private foundations and contributions from individuals”  <https://www.opensecrets.org/resources/faq>  List of funders with 1,664 entries, but no amounts /at least $5000).  <https://www.opensecrets.org/about/supporters> | <https://www.opensecrets.org> |
| S29 | USA | Tech Transparency Project (TTP) - Research initiative of Campaign for Accountability | NFP research NGO | “TTP is an information and research hub for journalists, academics, policymakers and members of the public interested in exploring the influence of the major technology platforms on politics, policy, and our lives.”  <https://www.techtransparencyproject.org/about-us>  Campaign for Accountability: “Our mission: Holding the powerful accountable. Campaign for Accountability is a 501(c)(3) nonpartisan, nonprofit watchdog organization that uses research, litigation, and aggressive communications to expose misconduct and malfeasance in public life. CfA works on behalf of the public interest to expose corruption, negligence, and unethical behavior wherever it may occur. We investigate the actions of powerful interests at every level of society, ranging from the largest corporations to the smallest county governments. Our current priorities include federal accountability, state oversight, corporate responsibility, and consumer protection.”  <https://campaignforaccountability.org/our-mission/> | TTP: “Our work is made possible by the generosity of individuals and foundations including Omidyar Network, Bohemian Foundation, Kapor Center, Craig Newmark Philanthropies, Skoll Foundation, and David Magerman. To ensure the independence of our work, TTP does not accept any donations from corporations.”  <https://www.techtransparencyproject.org/about-us>  Campaign for Accountability: Primarily funded through grants and donations. Major donors (for 2023) include: Warrett-Buffet Foundation, Fidelity Investments Charitable Gift Fund, New Venture Fund  <https://www.causeiq.com/organizations/campaign-for-accountability,814080431/> | <https://www.techtransparencyproject.org>  <https://campaignforaccountability.org> |
| S30 | USA | LobbyView |  | “LobbyView allows scholars, journalists, and citizens to learn more about lobbying in the U.S.”  <https://www.lobbyview.org/about/>  LobbyView is “a comprehensive lobbying database that is based on the universe of lobbying reports filed under the Lobbying Disclosure Act of 1995. LobbyView bridges two distinct observable political behaviors with regard to congressional bills: (1) sponsorship by politicians, and (2) reported lobbying by interest groups.”  <https://web.mit.edu/insong/www/pdf/lobbyview.pdf> | Website states “Supported by: Massachusetts Institute of Technology, National Science Foundation, Russel Sage Foundation.”  “∗Financial support from the National Science Foundation is acknowledged”.  <https://web.mit.edu/insong/www/pdf/lobbyview.pdf> | <https://www.lobbyview.org> |
| S31 | USA | The Intercept (project of First Look Media) | News publication, NFP organisation | “At The Intercept, we investigate powerful individuals and institutions to expose corruption and injustice. We see journalism as an instrument of civic action. We’re here to change the world, not just describe it.”  <https://theintercept.com/about/> | Relies on donations from readers.  <https://theintercept.com/about/>  Other funding includes $50 million at launch from eBay co-founder Pierre Omidyar  <https://www.influencewatch.org/non-profit/the-intercept/> | <https://theintercept.com> |
| S32 | USA | Sunlight Foundation | NFP organisation | Ceased active operations September 2020.  “The Sunlight Foundation is a national, nonpartisan, nonprofit organization that uses civic technologies, open data, policy analysis and journalism to make our government and politics more accountable and transparent to all.”  <https://sunlightfoundation.com/about/> | Funding page included with list of contributors (foundations and individuals) with amounts.  Includes $3.5 million contribution from co-founder Michael Klein.  <https://sunlightfoundation.com/about/funding/> | <https://sunlightfoundation.com> |
| S33 | USA | Project on Government Oversight | NFP watchdog NGO focused on government accountability | “Founded in 1981, the Project on Government Oversight (POGO) is a nonpartisan independent watchdog that champions good government reforms”  <https://www.pogo.org/about>  “We investigate corruption and abuse of power in the federal government. And we promote commonsense reforms that strengthen our democracy and build a more equitable, accountable government”.  <https://www.pogo.org> | “Accepts contributions from individuals and institutions.”  “To preserve our independence, POGO does not knowingly accept contributions from for-profit corporations, labor unions, any government, or anyone who stands to benefit financially from our work.”  <https://www.pogo.org/financials> | <https://www.pogo.org> |
| S34 | USA | CorpWatch (Project of the Social Good Fund) | Social Good Fund registered as NFP corporation | “Our mandate is to provide accurate, timely and easily accessible articles, reports and data on violations by multinational corporations to activists, media, the general public and policy makers.”  <https://www.corpwatch.org> | No current funding information for specifically Corpwatch.  Social Good Fund has Audited Financials document available for 2023 that includes list of grants (p.11).  <https://drive.google.com/file/d/1ZLgu-66Wtg-vgL0M7CNLLqsLXMy_1o9x/view> | <https://www.corpwatch.org> |
| S34 | USA | Global Exchange | NFP NGO for human rights and global justice | “Global Exchange is an international human rights organization dedicated to promoting social, economic, and environmental justice around the world. Defending workers, small farmers, vulnerable communities and the natural environment is at the heart of our mission.”  <https://globalexchange.org> | Funded by donations and memberships. Major donors include Rockefellar Brothers Fund, NDN Collective, Black Lives Matter Global Network Foundation  <https://www.influencewatch.org/non-profit/global-exchange/>  Financial statements available:  <https://globalexchange.org/wp-content/uploads/FS-fy-2020-and-2021.pdf> | <https://globalexchange.org> |
| S34 | USA | Public Citizen | NFP consumer rights advocacy NGO | “Public Citizen is a nonprofit consumer advocacy organization that champions the public interest in the halls of power. We defend democracy, resist corporate power, and fight to ensure that government works for the people – not big corporations”  <https://www.citizen.org/about/> | “We receive the majority of our funding from individual donors and foundation grants. We do not accept corporate or government money”. List of financial statements available.  <https://www.citizen.org/about/annual-report/> | <https://www.citizen.org> |
| S35 | USA | Wikipedia | NFP online encyclopaedia | “Wikipedia is a free online encyclopedia that anyone can edit, and millions already have. Wikipedia's purpose is to benefit readers by presenting information on all branches of knowledge. Wikipedia consists of freely editable content, with articles that usually contain numerous links guiding readers to more information.”  <https://en.wikipedia.org/wiki/Wikipedia:About> | “Wikipedia is hosted by the Wikimedia Foundation, a non-profit organization that also hosts a range of other projects.  The foundation finances itself mainly through millions of small donations from readers and editors, collected through email campaigns and annual fundraising banners placed on Wikipedia and its sister projects. These are complemented by grants from philanthropic organizations and tech companies, and starting in 2022, by services income from Wikimedia Enterprise.  <https://en.wikipedia.org/wiki/Wikimedia_Foundation> | <https://www.wikipedia.org> |
| S36 | USA | Bush School of Government and Public Service | Academic institution at Texas A&M University | “The Bush School of Government and Public Service was founded in 1997 under President George H. W. Bush’s philosophy that “public service is a noble calling.” Since then, the Bush School has continued to reflect that notion in curriculum, research, and student experience and has become a leading international affairs, political science, and public affairs institution.”  <https://bush.tamu.edu/about> | Funded by donations, state and university funding, and tuition paid by students.  <https://bush.tamu.edu/financialaid/>  Includes list of some funders (e.g. federal agencies), but no amounts.  <https://bush.tamu.edu/istpp/funders/> | <https://bush.tamu.edu> |
| S37 | USA | National Bureau of Economic Research | NFP research organisation | “The National Bureau of Economic Research (NBER) is a private, nonprofit organization that facilitates cutting-edge research on and analysis of major economic issues. It is nonpartisan and refrains from making policy recommendations, focusing instead on providing background studies and data that underlie decision-making in both the public and private sectors.”  <https://www.nber.org/about-nber> | “Research is supported by grants from government agencies or private foundations, by corporation and individual contributions, and by income from the NBER’s investment portfolio.”  Biggest funders - National Institute of Health, the National Science Foundation, the Social Security Administration, and the Alfred P. Sloan Foundation.  <https://www.nber.org/about-nber/support-funding> | <https://www.nber.org> |
| S38 | USA | Senator Elizabeth Warren | Individual (US Senator) | “Elizabeth Warren, a fearless consumer advocate who has made her life's work the fight for middle class families, was re-elected to the United States Senate for a second term on November 6, 2018, by the people of Massachusetts. Elizabeth is one of the nation's leading progressive voices, fighting for big structural change that would transform our economy and rebuild the middle class.”  <https://www.warren.senate.gov/about/about-elizabeth> | Funding of report unclear. Described as: “Prepared by Senator Elizabeth Warren”.  Contributors to her Campaign Committee Fundraising (2019-2024) outlined in OpenSecrets, including Massachusetts Institute of Technology, Google, Harvard University, Boston University, Apple.  <https://www.opensecrets.org/members-of-congress/elizabeth-warren/summary?cid=N00033492> | <https://www.warren.senate.gov> |
| S39 | USA | Congressional Research Service | Federal government agency | “The Congressional Research Service (CRS) serves as shared staff to congressional committees and Members of Congress.”  “CRS provides Congress with the vital analytical support it needs to address the most complex public policy issues facing the nation. Its work incorporates program and legislative expertise, quantitative methodologies and legal and economic analysis.”  The research divisions are:   - American Law - Domestic Social Policy - Foreign Affairs, Defense and Trade - Government and Finance - Resources, Science and Industry   CRS services come in many forms:   - reports on major policy issues - tailored confidential memoranda, briefings and consultations - seminars and workshops - expert congressional testimony - responses to individual inquiries   <https://www.loc.gov/crsinfo/about/> | Institute of US Congress (operates within Library of Congress) – “operates solely at the behest of and under the direction of Congress”  <https://www.congress.gov/crs-products> | <https://www.usa.gov/agencies/congressional-research-service> |
| S40 | USA | US Government Accountability Office (GAO) | Federal government agency for auditing and oversight. | “GAO, often called the "congressional watchdog,” is an independent, non-partisan agency that works for Congress. GAO examines how taxpayer dollars are spent and provides Congress and federal agencies with objective, non-partisan, fact-based information to help the government save money and work more efficiently.”  <https://www.gao.gov/about> | Works for US Congress. “Our work is done at the request of congressional committees or subcommittees or is statutorily required by public laws or committee reports, per our Congressional Protocols.”  <https://www.gao.gov/about/what-gao-does>  There are a number of checks in place to ensure GAO’s work remains independent and non-partisan.  <https://www.gao.gov/blog/gao-nonpartisan-heres-how-we-do-it> | <https://www.gao.gov> |
| S41 | Norway | U4 Anti-Corruption Resource Centre (hosted by Norwegian School of Business) | NFP research centre NGO | “At U4, we study the causes and pathways of corruption in society, document its effects on development outcomes, and identify and support efforts to address it.”  <https://www.u4.no/about-u4> | Permanent centre at Chr. Michelsen Institute, Norway (NFP). “U4's partners are likeminded agencies whose goal is to make lives better. Our task is to help the partners achieve results through effective anti-corruption efforts.  The bilateral international development agencies and foreign ministries on this page take part in the U4 Anti-Corruption Resource Centre partnership, and provide funding. We currently have partners in eight countries: Canada, Denmark, Finland, Germany, Norway, Sweden, Switzerland, and the United Kingdom.”  <https://www.u4.no/u4-partner-agencies> | <https://www.u4.no> |

1. **Challenges and recommendations**

| **Ref #** | **Organisation** | **Title** | **Challenges** | **Recommendations** |
| --- | --- | --- | --- | --- |
| S1 | Michael West Media | Revolving Doors – Democracy at risk | Not included in database. | Not included in database. |
| S2 | Centre for Public Integrity | The Lobbyist Register | Not included in database. | Not included within register section. Outlines that the register is its first step in creating a public resource that brings together data on the revolving door, political donations, lobbyist disclosures, registers of gifts, and policy outcomes. |
| S3 | Crikey | The gravy train list: what powerful politicians do for a living post-Parliament | Not outlined. | Introducing minimum times ex-politicians have to wait until accepting private sector employment. |
| S4 | Australia Institute & Australian Conservation Foundation | Greasing the Wheels: The systemic weaknesses that allow undue influence by mining companies on government: a QLD case study | Lack of transparency in lobbying. Very few lobbyists in QLD covered by the lobbying register. | Transparency - disclose employees’ industry connections. Tighten/regulate post-separation controls. Special Commission of Inquiry – investigate mining influence in QLD. Regulate lobbyists – include in-house lobbyists and increase transparency. Expand commission powers – strengthen crime and corruption commission. |
| S5 | Our Democracy Alliance (Australian Conservation Foundation, the Human Rights Law Centre and the Australian Democracy Network) | Explainer: It's time to shine a light on the shady world of political lobbying | Regulation of professional lobbying is weak. Weak disclosure laws (e.g. ministerial diaries). Insufficient requirements for Federal Register of Lobbyists (only about 20% lobbyists covered, do not have to disclose who they’re meeting, how often, what about). Cooling-off periods not enforced in practice. Lobbyists have access to orange passes granting access to Parliament House (access not afforded to voters). | Extend federal Register for Lobbyists to include in-house lobbyists. Regular disclosure – mandate reporting of lobbying activities. Strengthen enforcement – enhance the lobbyist code of conduct. Publish diaries – require ministers, shadow ministers, and senior staff to share their schedules. Prohibit industry job moves – prevent ministers from transitioning to industry roles post-tenure. |
| S6 | Greenpeace & Michael West | Dirty Power | Coal’s position in networks of influence enables its interests to remain central to the agenda of the current federal government. Former coal industry staff now enjoy direct access to senior government ministers. | Not the focus. Includes quote from Centre for Public Integrity that we require ‘greater regulation of political donations, lobbyists and the movement of staff between government and industry’. |
| S7 | The Conversation | Revealed: the extent of job-swapping between public servants and fossil fuel lobbyists | Parliamentary code of conduct bans former ministers from lobbying the federal government for 18 months after leaving office (but isn't enforced). Unknown extent big business tries bend the will of the body politic by influencing the formation of government policy. | Laws to prevent unwanted political activities e.g. cooling-off periods. Stronger disclosure laws regarding campaign financing and political donations. Federal anti-corruption body with independent investigative powers. |
| S8 | The Klaxon | Lockheed Martin, Australian Government joined at hip | Legal for powerful corporations to gain access to decision-makers via senior revolving door appointments. RD can channel people in both directions. | Not included. |
| S9 | Centre for Public Integrity | Closing the revolving door: Corporate influence and the need for lobbying reform | Current post-employment separation period not sufficient to dilute influence and connections of the regulated person. Current Commonwealth Code only applies to Ministers or Parliamentary Secretaries, ministerial advisers, Defence Force Officers and senior public servants. MPs not captured by current definition (unlike other jurisdictions) | More effective regulation – enshrine Lobbying Code of Conduct in legislation. Broader lobbying definitions, including in-house lobbyists. Enhance transparency – requiring publication of ministerial diaries, and expand lobbyist register to include meeting dates and details. Ensure accountability by appointing well-resource regulator to oversee Code, independent of Attorney General’s Department. Extend the post-employment separation period for officials. |
| S10 | Mandarin & Crikey | The Mandarin and Crikey's 'revolving door' list: How power bleeds between politics and the Big Four | RD can blur lines between public and private sectors. Profit incentives that drive private consultancies are not always compatible with the obligations of government officials and MPs to act in the public interest | Not included. |
| S11 | Crikey | Advisory firms to big pharma: Where do staffers go after politics? | Less attention falls to staffers who leave politics (compared to politicians). | Not included. |
| S12 | 350 Perth | Captured State: The influence of the gas lobby on WA | Current cooling-off periods are flawed and ineffective. Monitoring and tracking of methane emissions in Australia frequently underreported - full extent of life cycle emissions of gas are unknown. | Reform Aboriginal Heritage Act to ensure traditional owners have free, prior, and informed consent; lift gag clauses. Publish ministerial diaries real-time. Ban fracking in WA. Establish 18-month cooling-off period for elected officials and staff. Require real-time donation reporting. Enforce gas project conditions to hold polluters accountable. Review offset requirements. |
| S13 | Australia Institute | Too close for comfort | Lobbyist registers include only “third-party lobbyists”. Companies can employ their own team of government relations and public affairs staff, known as 'in-house' lobbyists. Senior management and owners of companies can directly lobby ministers and senior civil servants through formal and informal meetings. Companies can join industry bodies that lobby on their behalf of their members. | Require a broad public inquiry (QLD). Inquiry should stretch beyond political donations and investigate the role of lobbyists on relevant decision- making processes. Investigate impacts of the ‘revolving door’ between mining interests and public offices. |
| S14 | Michael West Media | Revolving Doors: how the fossil fuel lobby has governments ensnared | Weak guidelines – existing rules ignored; stricter laws exist in Canada and Ireland. Inadequate disclosure – some jurisdictions lack public records of lobbyist visits. Inconsistent registries – often have omissions and inconsistencies. Lobbying records are heard to access and search. | Establish dedicated Anti-corruption. Enforce five-year cooling-off period. Strengthen disclosure laws, including for campaign financing and party donations. Exclude institutional funding from politics. Laws for political advertising accuracy to hold media accountable for inaccuracies. |
| S15 | Grattan Institute | Who's in the room: Access and influence in Australian politics | Australia vulnerable to policy capture. Many of the ‘risk factors’ for policy capture are present in the Australian system – incl. financial dependence, cosy relationships and lack of transparency in dealings between special interests and parliamentarians. | Improve transparency in policy making (Publish ministerial diaries, link lobbyists register to orange passes, improve visibility of political donations). Strengthen accountability of policy makers (Set standards to avoid conflicts of interest, codes of conduct should be independently administered, establish a federal integrity or anti-corruption body). Level the playing field (Cap political advertising expenditure, boost countervailing voices). |
| S16 | Sydney Morning Herald | Canberra Inc: 'The revolving door' of lobbyists shaping today's policies | Individuals who leave the lobby register – or were never on it to begin with – face no penalty. Lack of regulation and enforcement. Meetings with lobbyists not recorded. Weak lobbyist rules. In-house lobbyists not counted. | Not included. |
| S17 | The Guardian | In the family': majority of Australia's lobbyists are former political insiders | Lack of regulation – no oversights on movements. Weakly enforced cooling-off periods. Lack of scrutiny for in-house lobbyists – operate with minimal transparency in federal government. Limited lobbyist register – only flags certain officials, missing connections. | Not included. |
| S18 | Campaign Against Arms Trade | Political Influence Browser | Challenges' header included. Elaborates on topics e.g. linked issues of borders, policing, climate crisis, not focused specifically on RD. UK arms trade would not be possible without political and economic support from government. | Not included. Alternatives' header included (e.g. shifting from arms to renewables to create better jobs), however, focus is on arms trade generally rather than RD. |
| S19 | Advisory Committee on Business Appointments (ACOBA) | Appointments taken up by former Crown servants (2014-2015 to 2017-2018) | Not included. | Not included. Recommendations given within individual letters regarding specific roles and conditions, but not for RD activities on a broader level. |
| S20 | Powerbase (Public Interest Investigations) | Category: UK Revolving door | Not included | Not included. |
| S21 | Greenpeace (Unearthed) | Revealed: How the gas industry spent tens of millions of pounds lobbying UK & EU policymakers | Calculating the amount spent on lobbying by fossil fuel giants is nearly impossible, secretive industry. | Not included. |
| S22 | Private Eye | Public servants, private paydays: How ministers and mandarins make life after government pay – a Revolving Doors Special | Revolving door removes tension between the state and the private sector (with which it should deal objectively). Both sectors employ the same people, and they think the same way. No part of government now questions the market in public services such as health. | Implement outright or extended bans on ministers and mandarins working in sectors they used to regulate or develop policy for. |
| S23 | Global Justice Now (formerly World Development Movement) | Web of power: The UK government and the energy- finance complex fuelling climate change | Climate impact of the strength of the financial lobby not widely known. High-carbon economy. Big finance and big energy closely linked. Energy-finance-government nexus is allowing fossil fuel companies to push the planet into climate crisis. | Government must regulate the finance sector’s investment in fossil fuels and break the money-energy nexus. |
| S24 | High Pay Centre | The revolving door - and the corporate colonisation of UK politics | ACOBA has no power of sanction. Number problems as result of revolving door incl. | Abolition of ACOBA and replacement with a statutory body. Following OECD guidelines. Cooling-off periods. Prohibitions on using confidential information or lobbying. Monitoring of post-employment behaviour and sanctions for non-compliance. Process and clear criteria for considering appointments. Transparency of appointments, disclosures and justification. |
| S25 | Spinwatch - Public Interest Investigations | Access all areas: Westminster's (vast) fracking lobby exposed | Lobbying connections are not always listed in the Coalition’s official Register of Consultant Lobbyists. | Not included. |
| S26 | Spinwatch - Monitoring PR and Spin | Revolving door is unhealthy | Lobbying rules loosely and variously interpreted. | Not included. |
| S27 | Transparency International UK | Managing revolving door risks in Westminster | Current oversight of RD is highly deficient, including scope of the rules. Rules are not enforced in practice, lack of sanctions and ability to monitor. Reliance on media to deter wrongdoing. A lack of coordination and consistency of arrangements. ACOBA is not fit for purpose and needs fundamental reform. | 10 key recommendations. Firstly, recommendations which strengthen the scope of the existing rules – including extending lobbying restriction periods, creating lobbying register, prohibit former officials from giving lobbying advice. Secondly, those which outline a new enforcement body, including establishing statutory enforcement body, enable it to impose obligations, require published transparency. |
| S28 | Open Secrets | Revolving door overview | Concept of the RD open to interpretation. Definition of ‘lobbyist’ under Lobbying Disclosure Act overly narrow. | Not included. |
| S29 | Tech Transparency Project (TTP) | Crypto Industry Amasses Washington Insiders as Lobbying Blitz Intensifies | Until now, crypto industry has largely avoided regulation in the U.S. Boston Fed’s new rules prevent officials from owning individual stocks, but don’t mention virtual currencies. Cooling-off periods not enforced. | Not included. |
| S30 | LobbyView | Lobbyist Data | Not included. | Not included. |
| S31 | The Intercept & Campaign for Accountability | The Android administration: Google's remarkably close relationship with the Obama White House, in two charts | Know little about what Google wants and gets from the US government. Sort of co-dependency between the two, where Google is vertically integrated with the government: a true public-private partnership. Doesn't just lobby the White House for favours, also collaborates with officials. | Not included. |
| S32 | Sunlight Foundation & Open Secrets | All Cooled Off: As Congress Convenes, Former Colleagues Will Soon be Calling From K Street | Law allows former members to “aid or advise clients (other than foreign governments or foreign political parties) concerning how to lobby Congress” (as long as they don’t lobby themselves). Lobbying restrictions more 'spaghetti than steel'. Many loopholes in lobbying laws, including whether lobbying must be disclosed to the public. | Describes proposed bills that failed to pass or were weakened, that would have restricted lobbying further during cooling-off periods (e.g. Close the Revolving Door Act). |
| S33 | Project on Government Oversight (POGO) | Dangerous Liaisons: Revolving Door at Securities and Exchange Commission (SEC) Creates Risk of Regulatory Capture | RD blurs lines between one of the nation’s most important regulatory agencies and the interests it regulates. SEC has exempted certain senior employees from a “cooling off period” | Recommendations to mitigate RD around: Letting the public see where federal employees go after leaving government. Extend cooling off periods for employees who enter and leave the agency. Give the public more information about agency actions. Give the SEC the resources it needs. |
| S34 | CorpWatch, Global Exchange, Public Citizen (Collaborative Report) | Bechtel: Profiting from Destruction - Why the Corporate Invasion of Iraq Must be Stopped | Not included. | Recommendations for contract decision-making processes: Open bidding processes to public scrutiny. Prioritise ethical companies and exclude firms (e.g. Bechtel) with poor integrity records. Investigate corruption, including war profiteering ties with Bush administration. Keep essential services like water under community control. Challenge free trade agreements for US/Middle East for its harmful impact. Redirect taxpayer funds to humanitarian aid and basic services over military contracts |
| S35 | Wikipedia | Revolving door (politics) – United States | Lobbying loopholes as many regulations do not apply to high-level policy makers. | Not included. |
| S36 | Bush School of Government and Public Service | Lobbying After Federal Service: The Revolving Door, Shadow Lobbying, and Cooling Off Periods for Former Government Officials | Missing data. Availability of data is dependent on each government department. Cooling-off regulations not always adhered to. Many engage in lobbying activities, even if not registered as lobbyists. Health issues were most often lobbied. | Further research required on relationships between the RD and lobbying activities in the executive branch (incl. other presidential administrations). |
| S37 | National Bureau of Economic Research (NBER) | From revolving doors to regulatory capture? Evidence from patent examiners | Examiners regulate IP protection by deciding what the patent rights include – meaning these individuals have considerable discretion in decision-making (versus if teams decided). Social norms big part of how things occur – doesn’t always need a conspiracy – it can be as simple as wanting to ‘avoid aggravating an individual from whom they are about to ask an important favour.’ | Additional research (How and whether policy should address the risks described). |
| S38 | Senator Elizabeth Warren | Pentagon Alchemy: How Defense Officials Pass Through the Revolving Door and Peddle Brass for Gold | Current federal ethics laws for regulating RD are overly complex and insufficient to prevent conflicts of interest. Post-government employment restrictions are confusing (“tangled mess”) that prevent effective implementation and compliance. | Legislative overhaul to address RD. Enact Senator Warren’s DoD Ethics and Anti-Corruption Act that imposes four-year ban on contractors hiring DoD officials. Mandate detailed annual reports from defence contractors to DoD on former senior officials they have employed. Stock ownership ban for senior DoD officials in major defence contractors. Recusal standards for DoD employees - require they recuse themselves from matters affecting their former employer’s financial interest for four years. |
| S39 | Congressional Research Service | Executive Branch Service and the “Revolving Door” in Cabinet Departments | Little data available about the RD. Ongoing discussion of whether revolving door restrictions are positive or negative. | Congress could amend law to consolidate the administration and enforcement of conflict of interest and RD provisions (so administration/enforcement not spread among several entities). Amending 'cooling-off' periods. |
| S40 | U.S. Government Accountability Office (GAO) | Post-government employment restrictions: DOD Could Further Enhance Its Compliance Efforts Related to Former Employees Working for Defense Contractors | Post-government employment restrictions not always adhered to. Parent company often controls several separate business units, which may track and store employee data using different methods. Challenge to identify all former DOD employees. Information systems cannot always automatically filter, manual file review. | Potentially amend the Defense Federal Acquisition Regulation Supplement so that contractors are required to represent their employees’ compliance with the PGE lobbying restrictions. |
| S41 | U4 Anti-Corruption Resource Centre | The Revolving Door Indicator: Estimating the distortionary power of the revolving door | No attempts to collect and use data on connections to build an actionable and internationally comparable indicator of the distortions created by RD process. | Need to identify institutional configurations under which the RD is likely to cause economic distortions and set rules to control it. RDI (revolving door indicator) - proxy for the distortionary effects of the revolving door in regulated sectors and industries - could be a first step toward estimating distortionary power of the RD. This could allow countries to be compared – e.g. for progress in implementing safeguards against the conflicts of interest associated. |

1. **Units of analysis**

| **Ref #** | **Organisation** | **Title** | **Revolving door level** | **Unit of analysis (Individual level)** | **Number of units (Individual level)** | **Individuals named** | **Unit of analysis (Industry or company level)** | **Number of units and named industries/actors (Company or industry level)** | **Unit of analysis (Govern-ment level)** | **Number of units and named parties (Government level)** |
| --- | --- | --- | --- | --- | --- | --- | --- | --- | --- | --- |
| S1 | Michael West Media | Revolving Doors – Democracy at risk | Individual; Industry; Government | Number of revolvers | 81 | Yes | Number of revolvers per industry | Fossil fuels (41); Military (19); Finance (10); Games and Liquor (11) | Number of revolvers per political party | Labor, Fossils Fuels (12); Labor, Military (4); Liberal, Fossil Fuels (21); Liberal, Military (4); LNP, Fossils Fuels (4); Nationals, Fossils Fuels (4) |
| S2 | Centre for Public Integrity | The Lobbyist Register | Individual | Number of revolvers | 283 (calculated from 40% of 707 lobbyists) | Yes | - | - | - | - |
| S3 | Crikey | The gravy train list: what powerful politicians do for a living post-Parliament | Individual; Governmen | Number of revolvers | 14 + | Yes | - | - | Number of revolvers per political party | Labor Ministry (14); Coalition Ministry (14) |
| S4 | Australia Institute & Australian Conservation Foundation | Greasing the Wheels: The systemic weaknesses that allow undue influence by mining companies on government: a QLD case study | Individual | Number of revolvers | 19 | Yes | - | Santos (6); Flinders Group (1); Flinders Hyder (2); Double B Advisory (1); Quaysource (1); Rowland Pty Ltd (1); GHD (1); Arrow Energy (3); QGC (1); Ambre Energy (3); AECOM (1); Origin Energy (1) | - | - |
| S5 | Our Democracy Alliance (Australian Conservation Foundation, the Human Rights Law Centre and the Australian Democracy Network) | Explainer: It's time to shine a light on the shady world of political lobbying | Individual | Number of revolvers | 12 | Yes | - | Rio Tinto; Minerals Council Australia; Woodside; APPEA; Seven Group Holdings; Responsible Wagering Australia; Packers Consolidated Press; Crown Melbourne; Dyne Maritime and American Global Strategies; CMAX Communications; EY; JPG Advisory; Cornerstone Group; Burson Cohn and Wolfe | - | - |
| S6 | Greenpeace & Michael West | Dirty Power | Individual; Company; Industry | Number of revolvers | 42 | Yes | Number of companies or groups | 21 Industry groups: Minerals Council Australia; Australian Coal Association; APPEA; QLD Resources Council. Coal industry: Glencore; Mitsubishi Development; Adani; Hancock Prospecting; Whitehaven; Peadbody Energy; Exxon. Lobby groups: CT Group; Capital Hill Advisory; Bespoke Approach; SAS Group. Media: News Corp; The Australian; Courier Mail; Daily Telegraph; Herald Sun; Sky News. | - | Coalition government |
| S7 | The Conversation | Revealed: the extent of job-swapping between public servants and fossil fuel lobbyists | Company; Government | - | - | Partly (22 named) | Number companies. Number of revolvers employed per company | 41 companies total. Energy companies: AGL (7); Ambre Energy (1): Arrow Energy (3); BG Group (6); BP Australia (1); Caltex Australia (1); Ergon Australia (1); Metgasco (1); Origin (2); Santos (16); Shell Australia (4); Total Australia (2). Lobbying firms: Australian Public Affairs (2); Barton Deakin (1); Bespoke Approach (4); Capitol Hill Advisory (1); CMAX Advisory (1); ECG Advisory Solutions (3); Endevour Consulting (3); Enhance Corporate (1); GRA Cosway (5); Premier State (3); SAS Group (4); Statecraft (4); The Next Level (6). Peak bodies: Australian Coal Association (2); APPEA Australian Petroleum Producers and Exporters Association (13); Minerals Council of Australia (8); NSW Minerals Council (10); Queensland Minerals Council (2); Energy Networks Association Australia (2). Mining/resource companies: Alcoa (1); BHP Billiton (3); Centennial Coal (1); Cuaesta Coal (1); Peabody Coal (2); Rio Tinto (5); Whitehaven Coal (2); Woodside (3). | Number of corporate links by political party | Labor (39); Liberal (44); National (10); Liberal National (7); |
| S8 | The Klaxon | Lockheed Martin, Australian Government joined at hip | Individual; Company | Number of revolvers | 12 | Yes | Company case study | Lockheed Martin | - | - |
| S9 | Centre for Public Integrity | Closing the revolving door: Corporate influence and the need for lobbying reform | Individual | Number of revolvers | 11 | Yes | - | Raytheon Technologies; Australian Hotels Assoication; Hemmes Trading; Multiplex; Lendlease; Twinza Oil; Tamboran Resources; Airbus; Ampol; British American Tobacco; Minerals Council NSW; Elgas; | - | Liberal (9); Labour (1) |
| S10 | Mandarin & Crikey | The Mandarin and Crikey's 'revolving door' list: How power bleeds between politics and the Big Four | Individual; Company | Number of revolvers | 50 | Yes | Number of revolvers to/from each consultancy | EY (9); KPMG (18); Deloitte (12); PwC (11) | - | Liberal (11); Labor (5); Family First (1) |
| S11 | Crikey | Advisory firms to big pharma: Where do staffers go after politics? | Individual | Number of revolvers | 15 | Yes | - | Foster McCrone Ratcliff & Sims; Bastion S&GO; York Park Group; Crown Resorts; AstraZeneca; Anacta; Responsible Wagering Australia; Sympli; Brookline Advisory; | - | Labor (4), Liberal (1) |
| S12 | 350 Perth | Captured State: The influence of the gas lobby on WA | Individual; Company; Government | Number of revolvers | 60 | Yes | Number of companies or groups | Fossil Fuels (11): QCG; Cooper; Strike; Chevron; Seven Group; Equinor; Santos; Woodside; Shell; BHP; Whitehaven Coal. Lobbying/membership groups (9): APPEA, Hawker Brotton, Minerals Council Australia, Australian Industry Greenhouse Network, International Gas Union, CME WA, GRA Partners, Cannings Purple, QLD Resources Council | Number of links per political party | Fed Labor (6); WA Labor (5); WA Nationals (2); Fed Nationals (1); Fed Liberals (4); WA Liberals (5) |
| S13 | Australia Institute | Too close for comfort | Individual | Number of revolvers | 16 | Yes | - | GHD (1); Arrow Energy (2); QGC (2); Santos (4); Flinders Hyder (2); Queensland Exploration Council (1); Next Level Holdings/Milner Strategic Services (2); CMAX Communications (1) | - | - |
| S14 | Michael West Media | Revolving Doors: how the fossil fuel lobby has governments ensnared | Individual | Number of revolvers | 19 | Yes | - | Santos (5); AGL (3); Eastern Star Gas (1); Whitehaven Coal (1); Lakes Oil (1); Queensland Resources Council (1); Shell (2); Australian Energy Market Commission (1); APPEA (2); Minerals Council of Australia (1); Queensland Gas Company/BG Group (1); Caltex (2); Energy Networks Association (1); NSW Minerals Council (2); Total (1) | - | Labor (2); National (2); Liberal (2) |
| S15 | Grattan Institute | Who's in the room: Access and influence in Australian politics | Industry; Government |  |  | Partly (8 named Table 2.1) | Number of lobbyists (and share of total) who were former government representatives (years 2012, 2013, 2014, 2015, 2016, 2017, 2018) | 118(18%); 177(30%); 190(31%); 176(31%); 179(31%); 212(37%); 193(36%) | Percentage of federal ministers or assistant ministers that take on roles with 'special interests' (peak body, lobby firm, big business, consulting) after politics | 28% Labor; Coalition (Figure 2.6) |
| S16 | Sydney Morning Herald | Canberra Inc: 'The revolving door' of lobbyists shaping today's policies | Company | - | - | Partly | Number of revolvers employed per lobby company | Lobby firms: Hawker Britton (4); Capitol Hill Advisory (5); Gracosway (8); Cornerstone Group (1). | - | Political alignment of lobbying firms described. Barton Deakin (Liberal aligned); Hawker Britton (Labor aligned) |
| S17 | The Guardian | In the family': majority of Australia's lobbyists are former political insiders | Industry; Government | - | - | - | Percentage of registered lobbyists who were former government representatives (years 2012, 2013, 2014, 2015, 2016, 2017, 2018) | 25; 30; 32; 31; 31; 36; 37 (Estimates from graph) | Number of lobbyists by former government role (Categories overlap and are not mutually exclusive) | Total 255. Unknown/former gov. rep (75); federal political staffer (74); state political staffer (69); party official (22); federal public servant (20); state politician (14); state public servant (10); federal politician (10) |
| S18 | Campaign Against Arms Trade | Political Influence Browser | Individual; Company | Number of revolvers | 200 | Yes | Number of named companies or groups | Total 31. Aegis; Airbus Group; Augusta Westland; Babcock; BAE Systems; Bechtel; BMT Group; Capita; Cohort; DynCorp; Fluor; General Dynamics; GKN; Holdingham Group; KBR; JCB; Leonardo; Marshall; MBDA; Northrop Grumman; QinetiQ; Raytheon; Rolls-Royce; SBAC; Serco; Smiths Group; Spearfish; Thales; Ultra Electronics; Vosper Thorneycroft; VPS | - | - |
| S19 | Advisory Committee on Business Appointments (ACOBA) | Appointments taken up by former Crown servants (2014-2015 to 2017-2018) | Individual | Number of (announced or taken up) revolving door cases | 111 | Yes | - | - | - | - |
| S20 | Powerbase (Public Interest Investigations) | Category: Revolving door | Individual | Number of revolvers for UK category | 52 | Yes | - | - | - | - |
| S21 | Greenpeace (Unearthed) | Revealed: How the gas industry spent tens of millions of pounds lobbying UK & EU policymakers | Individual; Company | Number of revolvers | 14 | Yes | Number of revolvers employed per company | Shell (4); BG Group (2); Centrica/British Gas (4); Anglo American (1); BHP Billiton (2); Drax (1) | - | Conservative Party (3) |
| S22 | Private Eye | Public servants, private paydays: How ministers and mandarins make life after government pay – a Revolving Doors Special | Individual | Number of revolvers | 30 | Yes | - | G4S (4); Babcock (4); BAE Systems (3); Lockheed (5); Thales (2); JP Morgan; Zurich Financial Services; Global Counsel LLP; Pimco; A4E; Bridgepoint Capital; Circle Holdings; Bechtel; Bain & Co; MHP Communications; Morgan Stanley; Prudential; Phoenix Group; RBS; Deloitte, HSBC (3); BlackRock (2); Terrington Management; Airbus Defence; Raytheon; Babcock Marine; EY; Atos (2); McKinsey; London and Continental Railways; URS | - | - |
| S23 | Global Justice Now (formerly World Development Movement) | Web of power: The UK government and the energy- finance complex fuelling climate change | Individual; Company | Number of revolvers | 38 | Yes | Number of government links per financial or fossil fuel company | RBS (3); HSBC (5); Barclays (3); Lloyds (2); Standard Chartered (5); Prudential (4); Legal & General (3); Aviva (4); BP (2); Shell (1); BP Group (3); Tullow Oil (1); Rio Tinto (1); Xstrata (1); Anglo American (1); BHP Billiton (1) | - | Conservative Party (5) |
| S24 | High Pay Centre | The revolving door - and the corporate colonisation of UK politics | Individual | Number of revolvers | 17 | Yes | - | HSBC (9); KPMG (1); Tesco (1); Maximus (1); PwC (1); Paddy Power (1); Frontier Economics (1); Booz Allan (1); Arup (1); British Land (1); Prudential (1) | - | - |
| S25 | Spinwatch - Public Interest Investigations | Access all areas: Westminster's (vast) fracking lobby exposed | Individual; Company | Number of revolvers | 34 | Partly | Number of fracking companies employing lobbying firms with former members of government; Number of lobbying companies employing people with access to government | Fracking companies (15): Energy UK; Task Force on Shale Gas; Parliamentary Group on Unconventional Gas; iGas; Third Energy; UK Onshore Operators Group; Celtique Energy; Tamboran; Ineos; Cuadrilla; North West Energy Task Force; Shale Gas Europe; Centrica; Australian Petroleum Production Exploration Association; GDF Suez. Lobbying firms (14): Fishburn; Edelmann; Hill & Knowlton; MHP; Newgate Communications; Weber Shandwick; Burson-Marstellar; Hanover; PPS; Westbourne Communications; FTI Consulting; Bell Pottinger; Crosby Textor; Brevia. | - | Conservative Party; Labour Party; Lib Dems |
| S26 | Spinwatch - Monitoring PR and Spin | Revolving door is unhealthy | Individual | Number of former health minister revolvers | 10 | Yes | - | Oasis Healthcare, BUPA, GSK, Hanover, International Federation of Health Plans, 2020health, Bridgepoint Capital, Association of the British Pharmaceutical Industry, Alliance Boots, Cinven, Apax Partners, GE Healthcare | - | Conservative Party; Labour Party |
| S27 | Transparency International UK | Managing revolving door risks in Westminster | Industry | - | - | Partly (6 named in case studies) | Number of roles taken outside of government by industry which overlap with remit of former minister/official; Proportion of roles outside of government which have an overlap with the remit of the former minister or official, as a proportion of total number of roles taken up in that industry (%) | Number roles: 18 education, 19 healthcare, 19 energy, 26 finance, 27 technology, 39 defence, and 52 other. Prevalence roles: 17% other, 24% finance, 40% technology, 42% energy, 46% healthcare, 58% education, 82% (defence) Note: estimated from graph. | - | - |
| S28 | Open Secrets | Revolving door overview | Individual; Company; Industry; Government agency | Number revolvers 'Important Job Holders'; Number of former Congress revolvers | 1819; 495; | Yes | Percentage of revolving door lobbyists per industry. Also includes number of revolvers employed per lobbying firm or other organisations. | Internet (42%); Telecom Services (41%); Misc Defense (40%); Securites and Investment (40%); Air Transport (39%); Electronics Mfg & Equip (39%); Oil & Gas (39%); Insurance (39%); Automative (38%); Electric Utilities (38%); Maunfacturing & Distributing (38%); Health Services/HMOs (38%); Pharmaceuticals/Health Products (37%); Civil Servants/Public Officials (37%); Business Associations (37%); Hospitals/Nursing Homes (36%); Non-Profit Institutions (35%); Education (34%); Misc Issues (33%); Human Rights (33%) | Number & percentage of revolvers by government administration. Number & percentage of revolvers by federal agency. | Range between 2.33% (Biden) and 20.6% (W.Bush). Party affiliations included |
| S29 | Tech Transparency Project (TTP) | Crypto Industry Amasses Washington Insiders as Lobbying Blitz Intensifies | Individual; Government | Number of revolvers | 235 | Yes | - | Firms included in spreadsheet | Number of moves between government agencies that directly regulate the financial sector and companies involved in crypto (78). Number moves between Congress and crypto/blockchain companies (87) | Top gov agencies include: Treasury (31); SEC (28); CFTC (15); Comptroller of the Currency (5); Consumer Financial Protection Bureau (CFPB) (3) |
| S30 | LobbyView | Lobbyist Data | Individual | Number of revolvers | 5820 | Yes | - | - | - | Codes listed for government agencies individuals worked for. |
| S31 | The Intercept & Campaign for Accountability | The Android administration: Google's remarkably close relationship with the Obama White House, in two charts | Company; Government | - | - | No | Number of revolving door cases Google into government; Number of revolving door cases government to Google | 55; 197 Google | - | Obama White House/Democrats (very close to Google) |
| S32 | Sunlight Foundation & Open Secrets | All Cooled Off: As Congress Convenes, Former Colleagues Will Soon be Calling From K Street | Individual; Government | Number of individual revolving door cases | 42 (12 detailed) | Yes | - | - | - | Congress 2015 (lists whether individuals were from Senate or House) |
| S33 | Project on Government Oversight (POGO) | Dangerous Liaisons: Revolving Door at Securities and Exchange Commission (SEC) Creates Risk of Regulatory Capture | Individual; Company; Government | Number of disclosure statements filed (most active SEC Alumni) | Between 15-46 | 23 named | Top SEC alumni recruiters; Number of SEC Alumni employed by firm; | ACA Compliance Group (16); Wilmer Cutler Pickering Hale and Dorr LLP (16); Deloitte LLP (14); Ernst & Young (12); KPMG LLP (12); Morgan, Lewis & Bockius LLP (11); Dechert (8); Morrison & Foerster LLP (8); O’Melveny & Myers LLP (8); K&L Gates LLP (7); Sidley Austin LLP (7); Skadden, Arps, Slate, Meagher & Flom LLP (7); Public Company Accounting Oversight Board (6); Deutsche Bank AG (5); DLA Piper (5); Gibson, Dunn & Crutcher LLP (5); PricewaterhouseCoopers LLP (5); Venable LLP (5); Wilson Sonsini Goodrich & Rosati, P.C. (5) | Former Divisions and Offices incl. number of SEC Alumni and number Disclosure Statements filed | SEC Securities and Exchange Commission (Gov. Agency) case study. Top 5: Enforcement (172; 1032); Regional Offices (52; 213); Office of the Chief Accountant (47; 146); Corporation Finance (46; 236); Investment Management (32; 111) |
| S34 | CorpWatch, Global Exchange, Public Citizen (Collaborative Report) | Bechtel: Profiting from Destruction - Why the Corporate Invasion of Iraq Must be Stopped | Individual; Company | Number of revolvers | 10 | Yes | Company case study | Bechtel | - | Republican administration |
| S35 | Wikipedia | Revolving door (politics) – United States | Individual | Number of revolvers | 21 | Yes | - | - | - | SEC |
| S36 | Bush School of Government and Public Service | Lobbying After Federal Service: The Revolving Door, Shadow Lobbying, and Cooling Off Periods for Former Government Officials | Company; Industry; Government | - | - | - | Individuals engaged with consultancies before/after federal service. Most (to least) lobbied areas/industries | Increased employment with consultancies after federal service. Health, Energy, Transport, Budget, Agriculture, Education, Environment, Defense, Tax, Banks Named actors: Accenture, BAE Systems, Booz Allen Hamilton, IBM, Deloitte, ICF International | Percentage career paths after federal service (by department); Percentage lobbyist after executive branch employment | Agriculture (5% law/consultancy, 5% lobbying, 8% private); Commerce (12% law/consultancy, 11% lobbying, 13% private); Defense (5% law/consultancy, 2% lobbying, 13% private); Education (10% law/consultancy, 3% lobbying, 5% private); Energy (13% law/consultancy, 5% lobbying, 17% private); Health/Human Services (10% law/consultancy, 3% lobbying, 5% private); Homeland Security (18% law/consultancy, 15% lobbying, 15% private). 54% lobbyist after executive branch employment |
| S37 | National Bureau of Economic Research (NBER) | From revolving doors to regulatory capture? Evidence from patent examiners | Company; Government | - | - | - | Number of patent examiners hired (by law firms that frequently hire examiners) | Banner Witcoff (10); Birch Stewart (15); Buchanan Ingersoll (12); Cooley (8); Finnegan Henderson (48); Fish & Richardson (13); Fitzpatrick (9); Foley & Lardner (13); Greenblum (9); Harness Dickey (11); Harrity & Harrity (11); Hunton (10); Knobbe (8); Lee & Morse (11); McDermott (8); Morgan Lewis (9); Oblon McClelland (17); Oliff (13); Sterne Kessler (15); Sughrue (17); Townsend (merged) (8); Venable (8) | - | USPTO United States Patent and Trademark Office (Gov. Agency) case study |
| S38 | Senator Elizabeth Warren | Pentagon Alchemy: How Defense Officials Pass Through the Revolving Door and Peddle Brass for Gold | Company; Government | - | - | Few named examples | Number of revolving door hires per company (top 20 defense contractors) | Total 672 revolving door lobbyists across 20 companies. Lockheed Martin (53), Boeing (85), Raytheon Technologies (64), General Dynamics (57), Pfizer (73), Northrop Grumman (32), Humana (34), Moderna (11), L3Harris Technologies (21), Huntington Ingalls (40), Regeneron Pharmaceuticals (13), Analytic Services (9), Leidos Holdings (37), BAE Systems (23), Centene (28), Bechtel (11), General Electric (60), McKesson (18), Atlantic Diving Supply (2), Bell Boeing (0). | - | Department of Defense (case study of contractors) |
| S39 | Congressional Research Service | Executive Branch Service and the “Revolving Door” in Cabinet Departments | Government | - | - | - | - | - | Number of lobbyists (and %) registered after by administration; Percentage of Department appointees registered as lobbyists | Bush administration; Obama administration. Bush 2001-2004 (134, 9.5%), Bush 2005-2008 (94, 5%), Obama 2009-2012 (27, 2%); Obama 2013-2016 (24, 1.2%); Between 1% (Justice) and 18% (Commerce). Department highest proportion registered as lobbyist **before**(61% Department of Veterans Affairs ); Department highest proportion registered as lobbyist after (82% Department of Transport); Department highest proportion registered as lobbyist **before and after** (50% Department of Labor) |
| S40 | U.S. Government Accountability Office (GAO) | Post-government employment restrictions: DOD Could Further Enhance Its Compliance Efforts Related to Former Employees Working for Defense Contractors | Company/Industry; Government | - | - | - | Number of revolvers (former DOD personnel) employed by 14 defence Contractors | Total 1,718 former DoD senior or acquisition officials who left DOD service hired per contractor. Raytheon Technologies (315); Northrop Grumman Corporation (289); General Dynamics Corporation (287); Lockheed Martin Corporation (253); L3 Harris Corporation (168); The Boeing Company (160); BAE Systems, Inc. (108); Federal Express Corporation (48); General Atomics (38); General Electric Company (31); Atlantic Diving Supply, Inc. (<10); McKesson Corporation (<10); AmerisourceBergen Corporation (<10); Bechtel Corporation (<10) | - | Department of Defense (case study of contractors) |
| S41 | U4 Anti-Corruption Resource Centre | The Revolving Door Indicator: Estimating the distortionary power of the revolving door | Company | Number of revolvers | 88 | No | Number of revolvers per company Goldman Sachs, Citigroup, Fannie Mae | Goldman Sachs (37), Citigroup (26), Fannie Mae (25) | - | - |
